# Differences in rating of health related quality of life on the EQ-5D-5 L between ethnic groups

**DOI:** 10.1007/s11136-025-04082-y

**Published:** 2025-10-23

**Authors:** Juan Marcelo Virdis, Laura Anselmi, Matt Sutton

**Affiliations:** 1 Health Organisation, Policy and Economics (HOPE) Research Group, Centre for Primary Care and Health Services Research (FBMH), University of Manchester, Oxford Rd, Manchester, M13 9PL UK; 2 Centre for Health Economics, Monash Business School, Monash University, 900 Dandenong Rd, Caulfield East, Melbourne, VIC 3145 Australia

**Keywords:** Self-rated health, HOPIT model, Ethnic inequalities, EQ-5D-5L

## Abstract

**Purpose:**

To examine whether ratings on each of the five dimensions of the EQ-5D-5L vary across ethnic groups after adjusting for health conditions and other relevant covariates.

**Methods:**

We applied the Hierarchical Ordered Probit (HOPIT) model to the EQ-5D-5L responses of 2,642,805 participants in nine pooled cross-sectional waves of the General Practice Patient Survey in England. The model separated latent health, determined by health conditions, from reporting behaviour, which was assumed to be influenced by socio-demographic characteristics, recent healthcare use, and healthcare access.

**Results:**

We find systematic differences in rating behaviour between five ethnic groups. Across all five dimensions, respondents from White ethnic backgrounds were most likely to choose middle severity levels. Respondents from Asian and Black ethnic backgrounds were more likely than those from White backgrounds to report the less severe levels for Anxiety/Depression and Usual Activities. Respondents from Other ethnic backgrounds not classified within the four main categories were more likely to choose the lowest and highest severity levels for Anxiety/Depression.

**Conclusion:**

Our results suggest important heterogeneity in self-rating of health and highlights the importance of adjusting for reporting heterogeneity in applications of this measure. Where such adjustments are not feasible, alternative approaches such as sensitivity analyses to test the impact of differential reporting should be considered.

**Supplementary Information:**

The online version contains supplementary material available at 10.1007/s11136-025-04082-y.

## Abstract (plain language summary)

Self-rated health (also called self-assessed or self-perceived health) is widely used in social science research. It is often measured by asking individuals to rate their health on a five-point scale. Previous research has raised concerns that differences in how people from different ethnic backgrounds rate their health might affect how health inequalities are measured. We examined whether people from different ethnic groups rate the five dimensions of health-related quality of life in the EQ-5D-5L instrument, even when reporting similar morbidity levels. Our analysis, using data from England, shows that self-rating of health varies across ethnic groups. Although the size and direction of the differences varied across health dimensions and ethnic groups, Asian and Black ethnic groups were generally more likely to report a better health status and White ethnic group respondents were generally more likely to choose middle levels of the scale. These findings raise concerns about comparisons of subjective measures across ethnically diverse population groups.

**Supplementary Information:**

The online version contains supplementary material available at 10.1007/s11136-025-04082-y.

## Introduction

Variation in the rating of self-reported health measures raises concerns about the validity and comparability of this widely used measure across diverse population groups. Self-rated health (also referred to as self-assessed or self-perceived health) is a commonly used measure in social science research [1] and has proven valuable in various domains, such as predicting mortality [2] and forecasting health care utilization [3–5]. Though a simple five-point scale is often used for self-rated health [6, 7], more complex tools relying on self-rating like the EQ-5D for measuring health-related quality of life are also widely employed. The EQ-5D-5L is a standardised instrument developed by the EuroQol Group [8, 9]. It asks individuals to report on five dimensions of health-related quality of life: Mobility, Self-care, Usual Activities, Pain/Discomfort, and Anxiety/Depression, each rated on a five-level scale. Understanding individuals' reporting behaviour across different dimensions of health, as well as on general health, is essential for ensuring valid and comparable health assessments.

Differences in reporting behaviour can have important implications for population health measurement, health technology assessment, and mortality prediction [2–5]. Previous studies have found that these differences can be associated with past health care utilisation and a range of demographic and socioeconomic factors, with ethnicity identified as a particularly significant factor [10–23]. While most of this research focused on general self-rated health, less attention has been given to multidimensional assessment tools like the EQ-5D [14, 24–26]. Given the longstanding inequalities in health between ethnic groups in England [27, 28], this setting provides a relevant context for assessing heterogeneity in reporting behaviour across different ethnic groups.

Various strategies have been developed to disentangle latent health from reporting behaviour. Anchoring vignettes—a description of the level of health of a hypothetical person which respondents are asked to evaluate on the same scale [18]—have been widely used in previous studies. Nevertheless, the effectiveness of anchoring vignettes depends on the assumptions of vignette equivalence and response consistency and evidence has emerged indicating violations of these assumptions [10, 29–34].

An alternative involves the use of self-reported diagnosed conditions, often termed "semi-objective" or "quasi-objective" measures, as they reflect factual matters such as diagnoses by physicians, despite being self-reported [16, 35–38]. While some studies suggest that self-reported conditions may lack accuracy [20, 58, 59], others have proposed their use because they may be less influenced by perception compared to fully self-rated measures [17]. To address the challenge of capturing latent health from these self-reported measures, prior studies have employed the Hierarchical Ordered Probit Model [16, 36–38]. Our study utilises this model to obtain a latent health index by modelling it as a function of objective (or semi-objective) health indicators, with thresholds influenced by variables affecting rating behaviour.

We examine whether reporting on each of the five dimensions of the EQ-5D-5L differs across five ethnic groups after adjusting for available indicators of health conditions, using nine waves of a uniquely large, nationally-representative repeated cross-sectional general population survey in England with a sample size of over 2.6million respondents. Our results provide valuable insights into heterogeneity in the reporting of EQ-5D-5L, which has not been explored as extensively as other self-rated health measures.

## Data

We obtained data from the General Practice Patient Survey (GPPS) for England, a repeated cross-sectional survey conducted by IPSOS for NHS England to capture patients’ experiences at GP practices [39]. The survey has been administered by post since 2007 (with an option to answer the questionnaire online) to patients aged 18 and above who had been continuously registered with a GP practice for at least six months. Each wave comprised a new, independent sample and weights were provided to adjust for non-response rates. The GPPS questionnaire, Frequently Asked Questions and covering letter were available in Arabic, Bengali, Czech, English, French, Gujarati, Mandarin, Polish, Portuguese, Punjabi, Slovak, Somali, Turkish, Urdu and, since 2017, Spanish [40–43].

We used data from nine waves of the GPPS that contained information on EQ-5D questionnaire. The EQ-5D is a standardised, non-disease-specific instrument to assess health-related quality of life [8]. It asks respondents to rate their level of wellness across five dimensions (Mobility, Self-care, Usual Activities, Pain/Discomfort, and Anxiety/Depression) using a categorical scale with labelled response options. The number of levels within each dimension has changed over time and currently the EQ-5D-5L uses 5 levels [9]. Responses to the EQ-5D-5L questionnaire were available in nine waves from 2012 to 2017, totalling 4,377,614 observations. We used the responses to the EQ-5D-5L questionnaire as the primary outcomes, and we refer to the levels as 1, 2, 3, 4 and 5, with 1 representing absence of problems and 5 the most severe category. Description of the levels is presented in Table 1.

**Table 1:** Descriptive statistics

	Frequency (%)				
	Asian	Black	Mixed	Other	White
*Mobility*					
1 No problems in walking about	84.23	83.89	83.59	78.94	78.88
2 Slight problems in walking about	8.78	8.64	8.91	10.66	11.20
3 Moderate problems in walking about	4.14	4.38	4.28	5.44	5.94
4 Severe problems in walking about	2.48	2.64	2.80	4.03	3.52
5 Unable to walk about	0.37	0.46	0.42	0.93	0.46
*Self-Care*					
1 No problems washing or dressing	93.19	93.58	92.55	89.66	92.12
2 Slight problems washing or dressing	3.19	2.88	3.34	4.56	3.71
3 Moderate problems washing or dressing	2.11	2.22	2.66	2.96	2.86
4 Severe problems washing or dressing	1.06	0.96	0.99	1.84	0.96
5 Unable to wash or dress	0.45	0.35	0.46	0.97	0.34
*Usual* *activities*					
1 No problems doing usual activities	82.54	83.28	78.68	78.08	75.82
2 Slight problems doing usual activities	10.19	9.34	12.16	11.53	13.54
3 Moderate problems doing usual activities	4.34	4.44	5.50	5.54	6.66
4 Severe problems doing usual activities	2.16	2.24	2.69	3.43	2.96
5 Unable to do usual activities	0.76	0.70	0.97	1.43	1.01
*Pain/Discomfort*					
1 No pain or discomfort	56.93	56.53	56.15	52.43	51.93
2 Slight pain or discomfort	27.69	27.11	27.67	27.24	29.75
3 Moderate pain or discomfort	10.19	10.81	10.77	11.87	12.72
4 Severe pain or discomfort	4.10	4.49	4.09	6.41	4.47
5 Extreme pain or discomfort	1.08	1.07	1.32	2.06	1.12
*Anxiety/Depression*					
1 Not anxious or depressed	73.97	77.06	64.20	69.29	67.73
2 Slightly anxious or depressed	17.38	14.43	21.19	18.39	19.79
3 Moderately anxious or depressed	5.71	5.72	9.76	6.95	8.98
4 Severely anxious or depressed	1.97	1.93	3.12	3.41	2.35
5 Extremely anxious or depressed	0.98	0.86	1.72	1.95	1.15
*Long-term conditions*					
Alzheimer’s disease or dementia	0.18	0.12	0.13	0.57	0.14
Angina or long-term heart problem	2.33	1.24	1.51	2.67	3.76
Arthritis or long-term joint problem	6.20	6.46	6.44	7.60	11.22
Asthma or long-term chest problem	7.76	7.09	11.91	7.75	10.84
Blindness or severe visual impairment	0.54	0.71	0.54	0.82	0.51
Cancer in the last 5 years	1.06	1.30	1.55	1.56	3.05
Deafness or severe hearing impairment	1.09	0.72	1.09	1.35	2.54
Diabetes	11.05	8.72	5.06	10.17	6.30
Epilepsy	0.68	0.62	1.16	0.90	1.17
High blood pressure	13.01	19.95	9.95	14.97	16.98
Kidney or liver disease	1.89	1.62	1.19	2.60	1.42
Long-term back problem	7.82	8.13	8.21	11.72	9.67
Long-term mental health problem	2.43	3.29	7.35	4.22	5.53
Long-term neurological problem	1.26	1.40	2.11	1.85	2.10
Another long-term condition	8.66	8.36	12.52	9.92	13.91
*Sex*					
Male	54.78	50.62	47.70	57.20	49.34
Female	45.22	49.38	52.30	42.80	50.66
*Deprivation index*					
1st tercile	18.20	8.31	22.60	13.35	36.42
2nd tercile	30.44	22.29	31.00	27.54	34.40
3rd tercile	51.37	69.39	46.39	59.11	29.18
*Age group*					
25–34	34.07	25.24	38.84	30.81	19.33
35–44	31.48	30.16	28.15	30.40	19.86
45–54	18.38	27.98	20.51	20.79	23.47
55–64	10.91	11.97	8.83	11.96	20.27
65–74	5.15	4.66	3.67	6.04	17.07
Total (%)	6.15	2.65	0.78	2.24	88.18
Total weighted (%)	6.32	2.59	0.95	2.32	87.82

*Note.* Weighted estimations. Number of observations per ethnic groups: White, 2,330,333; Mixed or Multiple Blackground, 20,642; Asian, 162,558; Black, 70,106; Other, 59,166; Total, 2,642,805. The ethnic groups are defined as follows: "White" includes English, Welsh, Scottish, Northern Irish, British, Gypsy or Irish Traveler, and Any other White background. "Mixed or Multiple Ethnic Groups" consist of White and Black Caribbean, White and Asian, White and Black African, or Any other Mixed/Multiple ethnic background. "Asian" comprises Indian, Pakistani, Bangladeshi, Chinese, or Any other Asian ethnicity. "Black" includes African, Caribbean, or Any other Black/African/Caribbean background. "Other Ethnic Groups" include Arab or Any other ethnic group

We used two sets of variables to investigate heterogeneity in reporting of the EQ-5D-5L: variables measuring health status directly and ethnicity alongside other variables that may influence rating behaviour. The health status measures were obtained from responses to the question: "Which, if any, of the following medical conditions do you have?" Respondents could select any from a list of 15 long-term conditions (see Table 1). Respondents were also asked, 'Have your activities been limited today because you have recently become unwell or been injured?' The possible response options were: “Yes, limited a lot”; “Yes, limited a little”; and “No.” These conditions were reported before answering the EQ-5D-5L.

Variables assumed to affect rating behaviour included ethnic background, interactions of sex with five age groups (25 to 34, 35 to 44, 45 to 54, 55 to 64, and 65 to 74) and the multiple deprivation index tercile of the area where the patient lives. This multiple deprivation index is a measure that synthesizes deprivation in income, employment, education, health, crime, access to housing and services, and the living environment into a single index [44]. We included ethnic background as a set of five binary indicators for the categories available in the dataset: White ethnic group (English/Welsh/Scottish/Northern Irish/British; Gypsy or Irish Traveller; and Any other White background); Mixed or Multiple Background ethnic group (White and Black Caribbean; White and Asian; White and Black African; and Any other Mixed/multiple ethnic background); Asian ethnic group (Indian; Pakistani; Bangladeshi; Chinese; and Any other Asian ethnicity); Black ethnic group (African; Caribbean; and Any other Black/African/Caribbean background); Other ethnic group (Arab; and Any other ethnic group). In addition, we incorporated a set of variables related to recent use of health care and GP practice size (full list available in supplementary material).

We excluded individuals younger than 25 (due to low probability of long-term conditions) and older than 74 years to avoid potential cognitive issues [45]. After the exclusion of 876,210 observations for individuals younger than 25 or older than 74 and 858,599 observations with missing values, of the final data included 2,642,805 observations. Comparisons of the frequency of ethnic groups between the full dataset, the analysis dataset and the proportions of ethnic groups based on census data from 2011 and 2021 are available in the supplementary matterial.

## Methods

### Conceptual framework

**Fig. 1:**
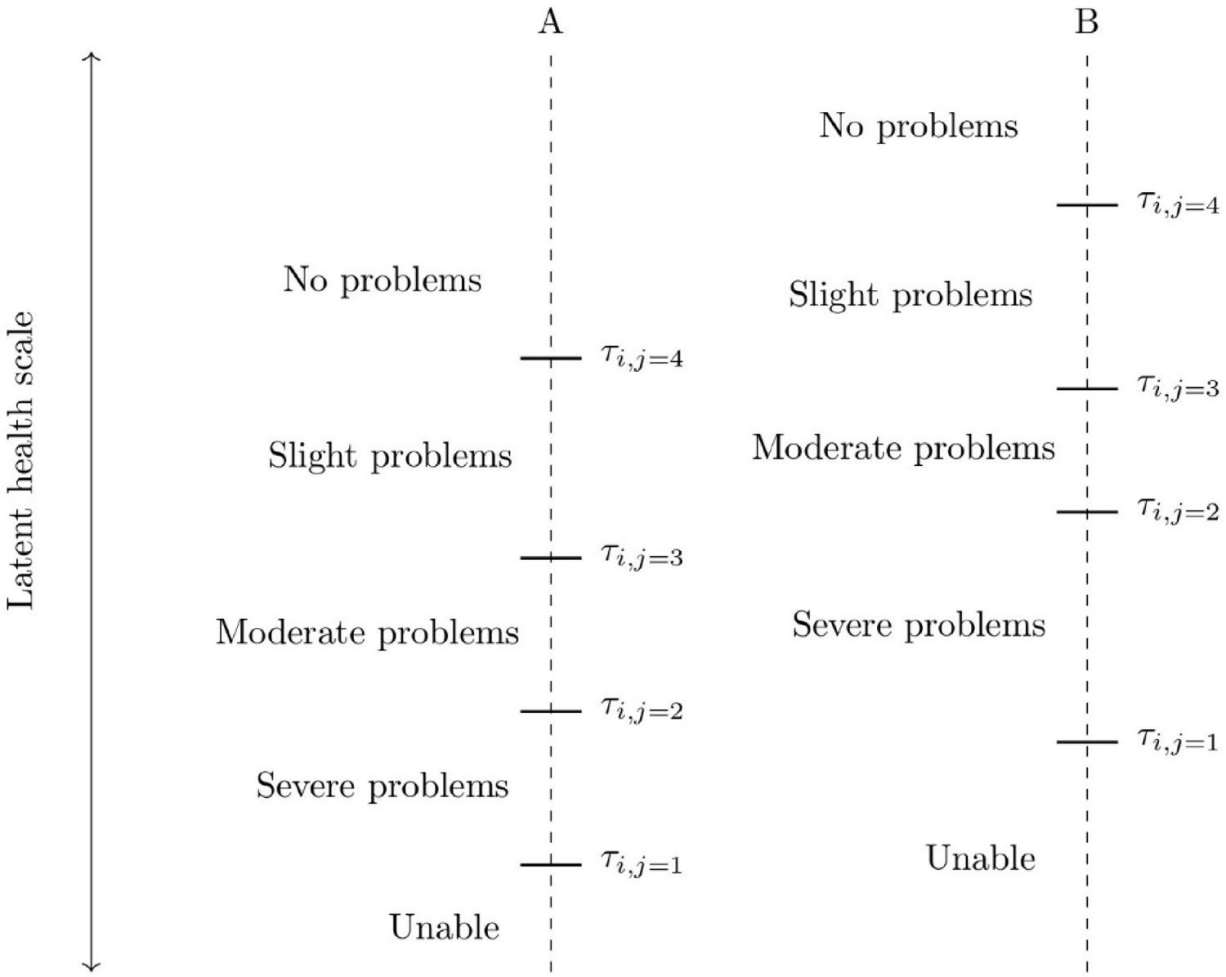
Illustration of cut-point differences for mobility scale for different population groups. *Note.* Based on Iburg et al. (2002). ${\tau }_{i,j}$ indicates threshold $j$ for individual $i$

We present the conceptual framework, based on Layes et al. [46]. We assume that there is a latent health variable which influences individuals’ health status rating and a set of thresholds which maps the latent health status into a discrete level of health. Figure 1 illustrates the interaction between latent status and thresholds. Consider groups A and B within the sample. If all individuals in group A share the same characteristics influencing thresholds, they will also share the same set of thresholds ${{\varvec{\tau}}}_{{\varvec{j}}}$, which may differ from the thresholds of group B, whose individuals possess a distinct set of characteristics. In the example shown in the diagram, a latent value that group B categorizes as 3 would be classified as 2 by group A. Depending on the latent value, the categorization between the groups may align or diverge.

### Empirical model

We applied the Hierarchical Ordered Probit (HOPIT) model [47, 48] separately for each of the five EQ-5D-5L dimensions, treating each dimension as an outcome. The latent health status ${h}_{i}^{*}$ is a function of a set of health-related variables ${{\varvec{x}}}_{{\varvec{i}}}$

1 $${h}_{i}^{*}={{\varvec{\beta}}}^{\boldsymbol{^{\prime}}}{{\varvec{x}}}_{{\varvec{i}}}+{\varepsilon }_{i}$$

where ${\varepsilon }_{i}$ is a normally distributed error term and ${\varvec{\beta}}$ is a set of coefficients related to the set of conditions ${{\varvec{x}}}_{{\varvec{i}}}$. The set of variables ${{\varvec{x}}}_{{\varvec{i}}}$ is hypothesized to affect an individual’s underlying health status and comprises binary morbidity variables and the interaction between two and three simultaneous conditions that were present in at least 1% of the sample [37].

Given a latent health value ${h}_{i}^{*}$, the individual $i$ will classify her health status into one of the $j=\{1,\dots ,j,\dots , 5\}$ categories, using a set of thresholds ${{\varvec{\tau}}}_{{\varvec{i}}{\varvec{j}}}$. Different parametrisations of the thresholds have been proposed in the literature [36, 47]. In this work, we followed Jürges' [36] parametrization which doesn’t allow variables to be in both equations, but ensures coherence of the thresholds as threshold values would be correctly ordered regardless of the values of the parameters:

2 $${h}_{i}=j, \text{if} \: {\tau }_{ij-1}\le {h}_{i}^{*}<{\tau }_{ij}$$

With

3 $$\begin{aligned} \tau_{ij} & = - \infty \quad \quad \quad \quad \quad \quad for\,j = 0 \\ \tau_{ij} & = \lambda_{j} + {\varvec{\gamma}}_{j} \user2{^{\prime}z}_{{\varvec{i}}} \quad \quad \quad for\,j = 1 \\ \tau_{ij} & = \tau_{i,j - 1} + e^{{\lambda_{j} + {\varvec{\gamma}}_{{\varvec{j}}} {\varvec{z}}_{{\varvec{i}}} }} \quad for\,j = 2 \ldots 4 \\ \tau_{i5} & = \infty \quad \quad \quad \quad \quad \quad \quad for\,j = 5 \\ \end{aligned} $$

where ${{\varvec{\gamma}}}_{{\varvec{j}}}$ is a set of coefficients for threshold ${\tau }_{j}$, and ${{\varvec{z}}}_{i}$ is the set of variables assumed to affect reporting behaviour and are not associated with the underlying health status given the set ${{\varvec{x}}}_{{\varvec{i}}}$ included in (1). The set ${{\varvec{z}}}_{{\varvec{i}}}$ was composed of a set of binary variables of age groups, sex, IMD, Ethnic group, use of GP services, use of Nurse services, ease to obtain an appointment and wave fixed effects.

### Allocation of variables and underlying assumptions

The HOPIT model explicitly separates variables into a latent health equation and a threshold equation, an approach related to the vignette method used to correct for reporting differences in health [10, 14, 16, 18, 21, 49, 50]. While vignettes rely on assumptions of vignette equivalence and response consistency, which may be violated [29–34], an alternative approach uses objective or semi-objective health conditions to account for true health status [16, 35, 36, 51]. Following this alternative, our study defined the latent health equation using 15 long-term conditions and limitations, including interactions to account for multimorbidity [37]. The threshold equation included variables previously associated with reporting behaviour, such as age, sex [10, 12, 19], economic deprivation [12], and recent healthcare use [17, 20]. We also included GP practice size and wave fixed effects to control for supply and time-related trends. Ethnic group was the key variable that we included in the threshold equation, to test its association with reporting behaviour.

### Estimation and empirical analysis

The sets of coefficients ${\varvec{\lambda}}$, ${\varvec{\gamma}}$ and ${\varvec{\beta}}$ are estimated by maximizing the following likelihood function

4 $$lnL={\sum }_{i=1}^{N}{\sum }_{j=1}^{5}{I}_{ij} \text{ln}\left[\Phi \left({\tau }_{ij}-{h}_{i}^{*}\right)-\Phi \left({\tau }_{ij-1}-{h}_{i}^{*}\right)\right]$$

where ${I}_{ij}=1$ if individual $i$ reported category $j$ and $\Phi$ is the cumulative distribution function (CDF) of the standard normal distribution. Survey weights were used for the estimation of the model.We focus primarily on the differences between ethnic groups. We examine the difference between the set of thresholds ${\varvec{\tau}}$ estimated for the White ethnic group, which is used as baseline, and for each of the remaining ethnic groups including Asian, Black, Mixed or Multiple Background and Other ethnic background. We proceed as follows. Let ${z}^{0}$(which are all binary variables) denote the set of variables where all are set to their sample proportions (the proportion of 1s in the sample), except for the ethnic group variables, which are set to zero to obtain fitted values for the baseline ethnic group (White ethnic group). Similarly, let ${z}^{1}$ represent the same set of variables, but with one of the ethnic group binary variables set to 1. The difference in thresholds between the baseline group and the group represented by the binary variable set to 1 can be expressed as:

5 $$ \begin{gathered} {\Delta }\tau_{j} = \lambda_{j} + {\varvec{\gamma}}_{{\varvec{j}}} \user2{^{\prime}z}^{1} - \left( {\lambda_{j} + {\varvec{\gamma}}_{{\varvec{j}}} \user2{^{\prime}z}^{0} } \right),\quad \quad for\;j = 1 \hfill \\ {\Delta }\tau_{j} = {\Delta }\tau_{j - 1} + e^{{\lambda_{j} + {\varvec{\gamma}}_{{\varvec{j}}} \user2{^{\prime}z}^{1} }} - e^{{\lambda_{j} + {\varvec{\gamma}}_{{\varvec{j}}} \user2{^{\prime}z}^{0} }} \quad \quad for\;j = 1 \ldots 4 \hfill \\ \end{gathered} $$

A positive difference suggests better self-ratings for the same latent health compared to respondents with White ethnic backgrounds. We assessed statistical significance by drawing 1,000 samples of coefficients, assuming a normal multivariate distribution and constructing 95% confidence intervals using both the percentile method and the bootstrapped standard error. In addition, we present the differences outlined in Eq. (5) using a faceted bubble plot.

Estimations were conducted using the hopit package [52] implemented in RStudio [53].

### Robustness checks

We estimated different model specifications. We first estimated a model with main effects of health conditions, ethnic groups and age-sex groups as explanatory variables. Next we added two- and three-way interactions of long-term conditions prevalent in at least 1% of the data. We then described how the difference in the thresholds defined in Eq. (5) change for these specifications compared to the full model. Tables A3 and A4, which present the results of these reduced models, are available in the supplementary material and the full list of coefficients can be found in the supplementary material. We also present in Tables A5 and A6 details on the number and percentage of missing values by variable and a linear probability model assessing the likelihood that an age-sex group, ethnic group, or health care usage category had a missing value in at least one of the other variables in the dataset. In addition, as to address potential relations of variables included in the threshold equation to health, we estimated three additional models including age and sex variables, area deprivation and health care utilization in set ${{\varvec{x}}}_{{\varvec{i}}}$ (latent health equation) and omitted from set ${{\varvec{z}}}_{{\varvec{i}}}$ (threshold equation). Finally, we conducted an analysis of heterogeneity within groups, presented in Tables A7–A11 in the supplementary material, by estimating the model for disaggregated ethnic groups.

## Results

Table 1 presents the descriptive statistics. The dataset included 2,642,805 individuals. Of these, 88.18% were of White ethnic background, 6.15% were Asian, 2.65% were Black, 0.95% were of Mixed or Multiple ethnic backgrounds, and 2.32% were categorized as Other. These percentages were similar to those in the full dataset but differed slightly from the overall population distribution in England based on census data. For example, the White ethnic group was overrepresented in our dataset compared to census data. Missing data analysis (Table A5) shows the highest proportion came from morbidity variables (9.53%). As missingness varied across observations and variables, the overall missing rate was 24.52%. Regression analysis (Table A6) revealed small but statistically significant differences in missingness by age, sex, deprivation, ethnicity, and healthcare use. Age effects were modest (max coefficient: 0.035), and males were 0.000 to 0.018 less likely to have missing values. The Other ethnic group showed the largest difference (0.156–0.160), followed by Black (0.062–0.063). Never having seen a GP was associated with a 0.302 increase in missingness. Adjusting for healthcare use had minimal effect on ethnic group differences (≤ 0.004).

In addition, Table 1 also displays the frequency of the five levels in each dimension of the EQ-5D-5L by ethnic group. The majority of responses indicated the absence of problems, particularly in Self-care, where proportions of the sample by ethnic group ranged from 89.66% to 93.58% reporting absence of problems. Some long-term conditions were highly prevalent, such as high blood pressure (affecting 9.95% to 19.95% of the sample) and arthritis (6.20% to 11.22%), while others were reported by only a small proportion of the sample, such as Alzheimer's disease or dementia (0.12% to 0.57%) and epilepsy (0.62% to 1.17%). Notably, between 8.36 and 13.91% of the individuals selected “Another long-term condition” in the questionnaire.

Table 2 presents the standardized coefficients associated with long-term conditions for each of the five models corresponding to the EQ-5D-5L dimensions. This analysis shows the level of association of each long-term condition with EQ-5D-5L dimensions. For instance, in the case of Anxiety/Depression, we found the highest coefficients for long-term mental health problems (0.35), Alzheimer's disease or dementia (0.09), and long-term neurological problems (0.11). A full list of coefficients, including two-way and three-way interaction terms, can be found in Table A2 of the supplementary material.

**Table 2:** Standardized coefficients (main effects^1^) and model fit

	Mobility	Self-care	Usual Activities	Pain/Discomfort	Anxiety/Depression
Alzheimer’s disease or dementia	0.046	0.130	0.104	-0.015	0.093
Angina or long-term heart problem	0.077	0.051	0.076	0.052	0.055
Arthritis or long-term joint problem	0.198	0.131	0.162	0.252	0.057
Asthma or long-term chest problem	0.038	0.024	0.036	0.016	0.024
Blindness or severe visual impairment	0.098	0.090	0.097	0.030	0.056
Cancer in the last 5 years	0.056	0.061	0.076	0.059	0.060
Deafness or severe hearing impairment	0.032	0.025	0.028	0.028	0.036
Diabetes	0.059	0.046	0.043	0.038	0.035
Epilepsy	0.080	0.101	0.075	0.021	0.049
High blood pressure	0.021	-0.004	0.010	0.012	0.022
Kidney or liver disease	0.053	0.044	0.048	0.058	0.045
Long-term back problem	0.160	0.121	0.158	0.257	0.074
Long-term mental health problem	0.077	0.124	0.151	0.055	0.350
Long-term neurological problem	0.182	0.159	0.161	0.176	0.110
Another long-term condition	0.099	0.093	0.106	0.124	0.086
Log-likelihood	− 1,449,143	− 710,246	− 1,593,384	− 2,372,822	− 2,126,040
AIC	2,898,740	1,420,945	3,187,223	4,746,097	4,252,533
Likelihood ratio index	0.346	0.355	0.310	0.259	0.128
Count R2	0.759	0.904	0.745	0.578	0.692

*Note*. Coefficients from HOPIT models. Survey weights were used for the estimation of the HOPIT model. The coefficients are standardized so that the highest latent health variable value equals 1 and the lowest equals 0. All coefficients included in the table are statistically significant (*p*-value < 0.001). The likelihood ratio index is calculated as one minus the ratio of the log-likelihood of the null probit model (including only the intercept) to that of the Hopit model. Count R^2^ represents the proportion of levels correctly classified

^1^The model also included health conditions representing limitations caused by recent injuries or short-term illnesses lasting a few days, as well as interactions between two and three conditions when these were prevalent in more than 1% of the sample. Coefficients and further details are provided in Table A2 of the supplementary material

**Fig. 2:**
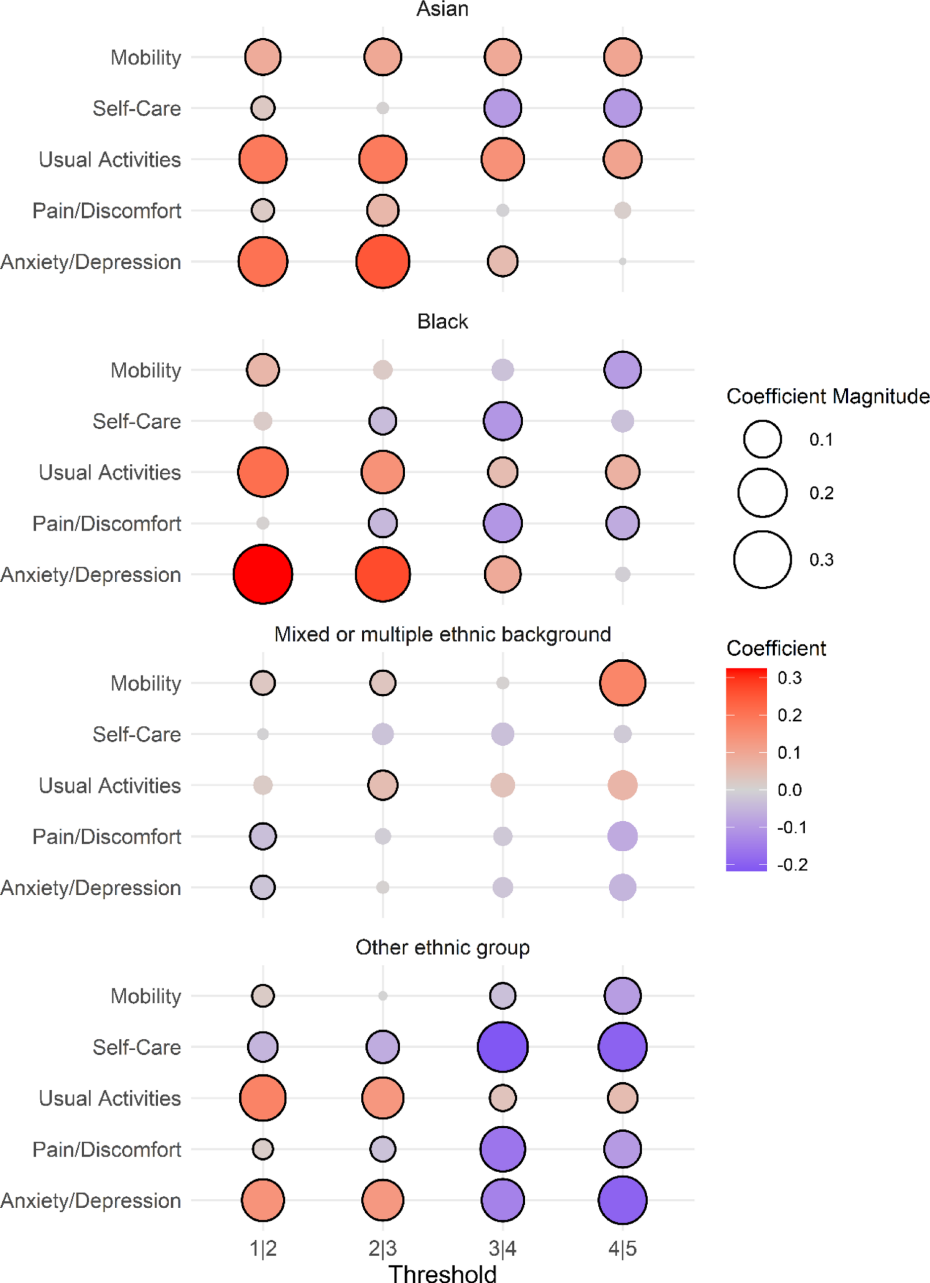
Differences in thresholds between each ethnic group and the White ethnic group for each severity level and EQ-5D domain. *Note.* A positive value indicates greater likelihood of reporting the less severe category for the same value of the latent health index. Solid black border indicates statistical significance using percentile bootstrapping (95% CI). Survey weights were used for the estimation of the HOPIT model

Figure 2 shows the differences in thresholds between categories by ethnic group, using the White ethnic group as baseline. The thresholds between levels 1 to 3 were predominantly positive for Asian and Black ethnic groups. This means that these groups were more likely to choose a less severe category given a fixed health status. However, this pattern was not observed for the Mixed or Multiple Background ethnic group, where the differences in thresholds were closer to zero and mostly non-significant, indicating similar rating behaviour to the White ethnic group. Additionally, larger positive differences, shown in red, appear in the thresholds between levels 1 and 3, whereas the largest negative differences, shown in blue, are found between levels 3 and 5.

These patterns are reflected in the numerical results presented in Table 3. For Asian and Black ethnic groups, similar results were found in the Anxiety/Depression dimension. These groups exhibited positive and statistically significant differences in thresholds between categories 1 to 4, with values between 0.054 and 0.269 (PV < 0.05), compared to the White ethnic group. Conversely, the difference in thresholds between categories 4 and 5 was not statistically significant. These two ethnic groups also showed similar results in the Usual Activities dimension, where the differences were between 0.053 and 0.212 (PV < 0.05). These findings suggest that Asian and Black ethnic groups were more likely than the White ethnic group to select less severe categories in the Usual Activities dimension, and in the Anxiety/Depression dimension, this tendency was observed only between levels 1 and 4.

**Table 3:** Differences in thresholds between each ethnic group and the White ethnic group baseline

Dimension	Thresholds between each of the levels			
	1|2	2|3	3|4	4|5
*Asian*				
Mobility	0.090* (0.005)	0.097* (0.007)	0.094* (0.010)	0.103* (0.02)
Self-Care	0.024* (0.007)	0.004 (0.010)	− 0.100* (0.013)	− 0.102* (0.020)
Usual Activities	0.191* (0.005)	0.190* (0.007)	0.145* (0.010)	0.107* (0.017)
Pain/Discomfort	0.021* (0.004)	0.063* (0.005)	− 0.004 (0.008)	0.013 (0.017)
Anxiety/Depression	0.206* (0.004)	0.251* (0.006)	0.054* (0.010)	0.000 (0.016)
*Black*				
Mobility	0.066* (0.010)	0.020 (0.014)	− 0.030 (0.020)	− 0.097* (0.034)
Self-Care	0.017 (0.014)	− 0.039* (0.018)	− 0.108* (0.025)	− 0.032 (0.039)
Usual Activities	0.212* (0.010)	0.144* (0.014)	0.053* (0.021)	0.076* (0.031)
Pain/Discomfort	0.004 (0.008)	− 0.046* (0.010)	− 0.108* (0.017)	− 0.070* (0.030)
Anxiety/Depression	0.324* (0.008)	0.269* (0.012)	0.092* (0.020)	− 0.008 (0.032)
*Mixed or multiple ethnic background*				
Mobility	0.027* (0.013)	0.031 (0.017)	0.004 (0.024)	0.169* (0.055)
Self-Care	− 0.003 (0.017)	− 0.029 (0.022)	− 0.033 (0.035)	− 0.015 (0.051)
Usual Activities	0.020 (0.012)	0.051* (0.015)	0.039 (0.023)	0.067 (0.036)
Pain/Discomfort	− 0.036* (0.009)	− 0.011 (0.013)	− 0.019 (0.020)	− 0.071 (0.037)
Anxiety/Depression	− 0.025* (0.009)	0.004 (0.012)	− 0.024 (0.021)	− 0.053 (0.031)
*Other ethnic background*				
Mobility	0.019* (0.009)	− 0.001 (0.011)	− 0.034* (0.015)	− 0.094* (0.026)
Self-Care	− 0.053* (0.012)	− 0.069* (0.015)	− 0.218* (0.020)	− 0.198* (0.028)
Usual Activities	0.174* (0.009)	0.134* (0.011)	0.036* (0.015)	0.053* (0.024)
Pain/Discomfort	0.015* (0.007)	− 0.031* (0.009)	− 0.165* (0.013)	− 0.100* (0.023)
Anxiety/Depression	0.141* (0.007)	0.130* (0.009)	− 0.143* (0.014)	− 0.196* (0.021)

*Note.* Bootstrap standard deviations are shown in parentheses. *Indicates statistical significance at the 95% confidence level (all results were significant using both parametric bootstrap standard deviations and the 2.5th and 97.5th percentiles). Survey weights were used for the estimation of the HOPIT model. The ethnic groups are defined as follows: "White" includes English, Welsh, Scottish, Northern Irish, British, Gypsy or Irish Traveler, and Any other White background. "Mixed or Multiple Ethnic Groups" consist of White and Black Caribbean, White and Asian, White and Black African, or Any other Mixed/Multiple ethnic background. "Asian" comprises Indian, Pakistani, Bangladeshi, Chinese, or Any other Asian ethnicity. "Black" includes African, Caribbean, or Any other Black/African/Caribbean background. "Other Ethnic Groups" include Arab or Any other ethnic group

The Other ethnic group showed positive and statistically significant differences with the white ethnic group in the threshold between categories 1 and 2 in the Mobility, Pain/Discomfort, and Anxiety/Depression dimensions, with values ranging from 0.019 and 0.174 (PV < 0.05). In contrast, the difference between thresholds for categories 4 and 5 ranged from − 0.085 and -0.196 (PV < 0.05). This indicates that individuals in the Other ethnic group were more likely than the White ethnic group to select the least severe category between levels 1 and 2, but more likely to select the most severe category between levels 4 and 5. A similar pattern was observed in other dimensions for different ethnic groups. For the Asian ethnic group, the threshold difference in the Self-Care dimension was 0.024 (PV < 0.05), while between levels 4 and 5 resulted − 0.102 (PV < 0.05). Similar results were found for the Black ethnic group in the Mobility dimension.

The sensitivity analysis shows significant changes when variables related to deprivation or health care use are omitted. As presented in table A4, the Asian ethnic group, which according to the results in Table 3 showed a positive difference compared to the White ethnic group between levels 1 and 2 across all dimensions, shifted to become negative and statistically significant for the Self-Care and Pain/Discomfort dimensions. Other cases of change of sign can be found for different dimensions and ethnic groups. When analysing the model presented in table A3, which differs from that of Table A4 by omitting interactions between long-term conditions, these omissions do not appear to have a substantial effect on the results. As shown in tables A5 to A7, results remain similar when variables related to age, sex and area deprivation were moved to the latent health equation, with slightly more negative changes for thresholds between levels 3 and 5 for ethnic groups other than the White. Tables A7-A11 in the supplementary material show results using more detailed ethnic groupings and reveal heterogeneity within the major ethnic groupings. For instance, within the White ethnic group, the Irish and Any other White background groups show positive changes in the threshold between levels 1 and 2, while the opposite was found for Gypsy or Irish Travelers. In the case of Asian ethnic groups, the Chinese consistently show a positive and statistically significant change between levels 1 and 4, which is not the case for other groups within the Asian category.

## Discussion

While widely used in social science, self-rated health measures are subject to concerns due to variation in how individuals assess their own health. In this study, we focused on the EQ-5D-5L, examining whether such variation exists across ethnic groups even after accounting for differences in health conditions. Using the White ethnic group as the comparator, we found that Asian, Black, and Other ethnic groups were generally more likely to report in each dimension the least severe of the levels 1 ("no problems"), 2 ("slight problems") and 3 ("moderate problems"), with a few exceptions. However, differences in self-rating behaviour are not uniform across all five levels. For instance, differences in Anxiety/Depression ratings between Asian and White ethnic groups were observed in the less severe categories, but not in the most severe ones. Moreover, heterogeneity was also found between more disaggregated ethnic groups.

Our findings are consistent with existing literature suggesting that reporting behaviours are often shaped by socio-cultural factors. For instance, Twaddle [54] and Smith et al. [55] highlighted that health and illness are often viewed within a sociological framework, where perceptions of “normal” health and deviations from it can differ significantly across population groups. Furthermore, McMullen and Luborsky [56] emphasise the role of comparative rationales, where individuals assess their health relative to expectations based on age, limitations, disease, ethnicity or gender. These studies provide a plausible interpretation of the variation in reporting behaviour across ethnic groups. We also found that self-perceived poor health was associated with health care utilisation, which is consistent with previous research [57].

Our study provides new evidence to support heterogeneity in self-rating of health in relation to ethnicity within a country in the case the EQ-5D-5L which has been subject to less research. Moreover, our uniquely large dataset enabled us to control for a comprehensive set of long-term conditions, allowing for a more robust assessment. In addition, our results in some of the dimensions suggest that, compared to White ethnic groups, the Other ethnic group is more likely to select middle levels of the ordinal scale. This finding is consistent with previous research in the United States comparing White ethnic groups with Hispanic and non-Hispanic Black ethnic groups [21].

The sensitivity analysis yielded interesting results regarding the impact of use of health care and deprivation. Notably, when we included variables related to GP and nurse visits, ease of obtaining an appointment and deprivation, results changed significantly. The results by ethnic group presented in Table 3 and the sensitivity analysis presented in Table A3 shows that part of the effect of deprivation and health care use variables was captured by ethnicity in the reduced model which included only age, sex and ethnic groups. This is consistent with previous studies which found differences in reporting health related to use of health care [17, 20]. Furthermore, our core finding regarding ethnic differences in selecting extreme reporting categories remained consistent across different model specifications, including when age, sex, deprivation, and health service use variables were re-specified within the latent health equations.

The interpretation of this study’s results must take the following limitations into account. First, the list of long-term conditions may only capture latent health partially. Second, other socio-economic and demographic variables could influence rating behaviour and omission of these variables might have induced bias into our results. Third, the long-term conditions considered in this study are self-reported and, therefore, may themselves be subject to reporting differences [58, 59]. Fourth, errors in reporting may be biased toward certain ethnic groups, meaning that some of our results might reflect this bias rather than genuine heterogeneity in rating behaviour. Finally, missing data may affect findings, particularly for the 'Other Ethnic' group, which had a significantly higher probability of missing values compared to the White ethnic group.

Further research using more comprehensive data on health conditions is needed to assess the robustness of our results. Access to objectively measured health conditions would help mitigate potential variations in reporting between population groups. Additionally, investigating the factors that individuals consider when answering health-related questions could provide valuable insights. Future work should also explore variation in reporting within broader ethnic categories. Our results suggest important heterogeneity in self-rating of health, and highlights the importance of adjusting for reporting heterogeneity in applications of this measure. Where such adjustments are not feasible, alternative approaches such as sensitivity analyses to test the impact of differential reporting should be considered.

## Supplementary Information

Below is the link to the electronic supplementary material.

Supplementary Material 1

Supplementary Material 2

## Data Availability

Individual-level data from GPPS were obtained from Ipsos MORI via an NHS England sharing agreement with our team at the University of Manchester.
