# Supplementary material for: Differences in rating of health related quality of life on the EQ-5D-5 L between ethnic groups
Source: Qual Life Res. 2025 Oct 23;34(11):3231–43. doi: 10.1007/s11136-025-04082-y (PMC12681471; doi:10.1007/s11136-025-04082-y)
Supplement: Supplementary file 2 — Supplementary Material 2 [file 11136_2025_4082_MOESM2_ESM.docx]

**Appendix**

| **Table A1.** Frequencies of ethnic groups in the dataset and comparison with census data | | | | | | | | |
| --- | --- | --- | --- | --- | --- | --- | --- | --- |
|  | Census data | | Original Dataset^1^ | | Analysis Dataset^2^ | | |  |
| Ethnic Group | 2011 (%) | 2021 (%) | Simple (%) | Weighted (%) | Simple (%) | Weighted (%) | |  |
| Asian | 7.51 | 9.25 | 6.00 | 6.33 | 6.15 | 6.32 | |  |
| Black | 3.33 | 4.04 | 2.68 | 2.65 | 2.65 | 2.59 | |  |
| Mixed or Multiple Background | 2.18 | 2.88 | 0.73 | 0.90 | 0.78 | 0.95 | |  |
| White | 85.97 | 81.71 | 87.97 | 87.35 | 88.18 | 87.82 | |  |
| Other | 1.01 | 2.11 | 2.62 | 2.76 | 2.24 | 2.32 | |  |
| *Note.* Source: Office for National Statistics – Census 2021 and own elaboration based on GPPS survey. The ethnic groups in are defined as follows: "White" includes English, Welsh, Scottish, Northern Irish, British, Gypsy or Irish Traveler, and Any other White background. "Mixed or Multiple Ethnic Groups" consist of White and Black Caribbean, White and Asian, White and Black African, or Any other Mixed/Multiple ethnic background. "Asian" comprises Indian, Pakistani, Bangladeshi, Chinese, or Any other Asian ethnicity. "Black" includes African, Caribbean, or Any other Black/African/Caribbean background. "Other Ethnic Groups" include Arab or Any other ethnic group.  ^1^Percentages from the original datasets are based on all available data from waves "Y7W1", "Y7W2", "Y8W1", "Y8W2", "Y9W1", "Y9W2", "Y10W1", "Y10W2", and "Y11" (n = 4,207,283). 2.02% didn’t have ethnic group information.  ^2^Based on dataset used in the descriptive statistics and estimations (n = 2,642,805). | | | | | | |  |  |

| **Table A2 (Part 1 of 3).** Standardized coefficients for health conditions: main effects and interactions (Main Model) | | | | | |  |
| --- | --- | --- | --- | --- | --- | --- |
| Condition | Mobility | Self-care | Usual Activities | Pain / Discomfort | Anxiety / Depression | |
| Alzheimer’s disease or dementia | 0.046 | 0.13 | 0.104 | -0.015 | 0.093 | |
| Angina or long-term heart problem | 0.077 | 0.051 | 0.076 | 0.052 | 0.055 | |
| Arthritis or long-term joint problem | 0.198 | 0.131 | 0.162 | 0.252 | 0.057 | |
| Asthma or long-term chest problem | 0.038 | 0.024 | 0.036 | 0.016 | 0.024 | |
| Blindness or severe visual impairment | 0.098 | 0.09 | 0.097 | 0.03 | 0.056 | |
| Cancer in the last 5 years | 0.056 | 0.061 | 0.076 | 0.059 | 0.06 | |
| Deafness or severe hearing impairment | 0.032 | 0.025 | 0.028 | 0.028 | 0.036 | |
| Diabetes | 0.059 | 0.046 | 0.043 | 0.038 | 0.035 | |
| Epilepsy | 0.08 | 0.101 | 0.075 | 0.021 | 0.049 | |
| High blood pressure | 0.021 | -0.004 | 0.01 | 0.012 | 0.022 | |
| Kidney or liver disease | 0.053 | 0.044 | 0.048 | 0.058 | 0.045 | |
| Long-term back problem | 0.16 | 0.121 | 0.158 | 0.257 | 0.074 | |
| Long-term mental health problem | 0.077 | 0.124 | 0.151 | 0.055 | 0.35 | |
| Long-term neurological problem | 0.182 | 0.159 | 0.161 | 0.176 | 0.11 | |
| Another long-term condition | 0.099 | 0.093 | 0.106 | 0.124 | 0.086 | |
| Recent Illness/Injury Limitations^1^: Severe (Baseline: None) | 0.069 | 0.036 | 0.074 | 0.104 | 0.06 | |
| Recent Illness/Injury Limitations^1^: Moderate (Baseline: None) | 0.156 | 0.132 | 0.161 | 0.184 | 0.12 | |
| *Note*. The coefficients from the model are standardized so that the highest latent variable value equals 1 and the lowest equals 0. Survey weights were used for the estimation of the HOPIT model.  ^1^Respondents were instructed to consider injuries or health problems lasting "a few days or weeks." | | | | | |  |

| **Table A2 (Part 2 of 3).** Standardized coefficients for health conditions: main effects and interactions (Main Model) | | | | | |  |
| --- | --- | --- | --- | --- | --- | --- |
| Condition | Mobility | Self-care | Usual Activities | Pain / Discomfort | Anxiety / Depression | |
| Angina or long-term heart problem and Arthritis or long-term joint problem | -0.051 | -0.027 | -0.049 | -0.034 | -0.018 | |
| Angina or long-term heart problem and Diabetes | -0.002 | -0.007 | -0.006 | 0.003 | -0.013 | |
| Angina or long-term heart problem and High blood pressure | -0.005 | 0.001 | -0.004 | 0.003 | -0.003 | |
| Arthritis or long-term joint problem and Asthma or long-term chest problem | -0.003 | 0.012 | -0.003 | 0.008 | 0.01 | |
| Arthritis or long-term joint problem and Deafness or severe hearing impairment | -0.034 | -0.023 | -0.031 | -0.027 | -0.017 | |
| Arthritis or long-term joint problem and Diabetes | -0.014 | -0.004 | -0.01 | -0.002 | 0.008 | |
| Arthritis or long-term joint problem and High blood pressure | 0.002 | 0.014 | 0.005 | 0.006 | 0.003 | |
| Arthritis or long-term joint problem and Long-term back problem | -0.088 | -0.038 | -0.072 | -0.143 | 0.006 | |
| Arthritis or long-term joint problem and Long-term mental health problem | -0.034 | -0.05 | -0.073 | -0.009 | -0.04 | |
| Arthritis or long-term joint problem and Another long-term condition | -0.049 | -0.035 | -0.046 | -0.059 | -0.017 | |
| Asthma or long-term chest problem and Diabetes | -0.001 | -0.005 | -0.005 | 0.005 | -0.003 | |
| Asthma or long-term chest problem and High blood pressure | 0.02 | 0.024 | 0.019 | 0.023 | 0.01 | |
| Asthma or long-term chest problem and Long-term back problem | -0.016 | -0.015 | -0.022 | -0.011 | -0.007 | |
| Asthma or long-term chest problem and Another long-term condition | -0.013 | -0.01 | -0.016 | 0.001 | -0.011 | |
| Cancer in the last 5 years and High blood pressure | -0.032 | -0.038 | -0.04 | -0.029 | -0.035 | |
| Deafness or severe hearing impairment and High blood pressure | -0.006 | -0.006 | -0.004 | 0.002 | -0.012 | |
| Diabetes and High blood pressure | 0.004 | 0.009 | 0.007 | 0.009 | -0.002 | |
| Diabetes and Long-term back problem | -0.007 | -0.008 | -0.013 | -0.004 | 0.001 | |
| Diabetes and Another long-term condition | 0.007 | 0.004 | -0.001 | 0.001 | -0.004 | |
| *Note*. The coefficients from the model are standardized so that the highest latent variable value equals 1 and the lowest equals 0. Survey weights were used for the estimation of the HOPIT model.  ^1^Respondents were instructed to consider injuries or health problems lasting "a few days or weeks." | | | | | |  |

| **Table A2 (Part 3 of 3).** Standardized coefficients for health conditions: main effects and interactions (Main Model) | | | | | |  |
| --- | --- | --- | --- | --- | --- | --- |
| Condition | Mobility | Self-care | Usual Activities | Pain / Discomfort | Anxiety / Depression | |
| High blood pressure and Long-term back problem | -0.011 | 0.005 | -0.011 | -0.01 | 0.006 | |
| High blood pressure and Another long-term condition | -0.008 | -0.001 | -0.01 | -0.013 | -0.018 | |
| Long-term back problem and Long-term mental health problem | -0.024 | -0.046 | -0.074 | -0.004 | -0.054 | |
| Long-term back problem and Another long-term condition | -0.037 | -0.035 | -0.045 | -0.053 | -0.02 | |
| Arthritis, Asthma, and High blood pressure | -0.028 | -0.031 | -0.025 | -0.031 | -0.019 | |
| Arthritis, Diabetes, and High blood pressure | -0.016 | -0.018 | -0.012 | -0.022 | -0.014 | |
| Arthritis, High blood pressure, and Long-term back problem | 0.013 | -0.004 | 0.012 | 0.016 | -0.009 | |
| *Note*. The coefficients from the model are standardized so that the highest latent variable value equals 1 and the lowest equals 0. Survey weights were used for the estimation of the HOPIT model.  ^1^Respondents were instructed to consider injuries or health problems lasting "a few days or weeks." | | | | | | |

| **Table A3**. Sensitivity Analysis – Differences in Thresholds Between Ethnic Groups and the White Ethnic Group Baseline (Omission of interactions of long-term conditions in latent equation, and area deprivation and health care use variables in the threshold equations ^1^) | | | | |
| --- | --- | --- | --- | --- |
| Dimension | Threshold | | | |
|  | 1\|2 | 2\|3 | 3\|4 | 4\|5 |
| Asian | | | | |
| Mobility | 0.016* (0.004) | 0.031* (0.006) | 0.036* (0.008) | 0.121* (0.018) |
| Self-Care | -0.057* (0.006) | -0.056* (0.008) | -0.168* (0.011) | -0.133* (0.017) |
| Usual Activities | 0.118* (0.004) | 0.119* (0.006) | 0.07* (0.009) | 0.093* (0.014) |
| Pain/Discomfort | -0.037* (0.003) | -0.023* (0.005) | -0.102* (0.007) | -0.073* (0.013) |
| Anxiety/Depression | 0.16* (0.004) | 0.191* (0.005) | -0.026* (0.008) | -0.078* (0.012) |
| Black | | | | |
| Mobility | 0.003 (0.007) | -0.027* (0.009) | -0.04* (0.013) | -0.068* (0.026) |
| Self-Care | -0.032* (0.009) | -0.065* (0.012) | -0.129* (0.017) | -0.106* (0.026) |
| Usual Activities | 0.151* (0.007) | 0.09* (0.009) | 0.032* (0.012) | 0.062* (0.021) |
| Pain/Discomfort | -0.036* (0.005) | -0.087* (0.007) | -0.17* (0.01) | -0.075* (0.021) |
| Anxiety/Depression | 0.277* (0.006) | 0.209* (0.008) | 0.019 (0.012) | -0.011 (0.02) |
| Mixed or multiple ethnic background | | | | |
| Mobility | -0.025* (0.011) | -0.022 (0.015) | -0.024 (0.021) | 0.149* (0.048) |
| Self-Care | -0.071* (0.014) | -0.079* (0.018) | -0.061* (0.028) | -0.055 (0.043) |
| Usual Activities | -0.021* (0.01) | 0.012 (0.014) | 0.004 (0.02) | 0.02 (0.032) |
| Pain/Discomfort | -0.063* (0.009) | -0.048* (0.012) | -0.077* (0.018) | -0.085* (0.032) |
| Anxiety/Depression | -0.054* (0.009) | -0.026* (0.011) | -0.071* (0.017) | -0.09* (0.025) |
| Other ethnic background | | | | |
| Mobility | -0.073* (0.007) | -0.079* (0.009) | -0.133* (0.011) | -0.12* (0.022) |
| Self-Care | -0.162* (0.008) | -0.151* (0.01) | -0.313* (0.013) | -0.294* (0.02) |
| Usual Activities | 0.097* (0.007) | 0.068* (0.009) | -0.034* (0.012) | -0.036 (0.018) |
| Pain/Discomfort | -0.041* (0.006) | -0.103* (0.007) | -0.26* (0.01) | -0.217* (0.016) |
| Anxiety/Depression | 0.1* (0.006) | 0.085* (0.007) | -0.216* (0.011) | -0.248* (0.015) |
|  |  |  |  |  |
| *Note.* Bootstrap standard deviations are shown in parentheses. *Indicates statistical significance at the 95% confidence level (all results were significant using both parametric bootstrap standard deviations and the 2.5th and 97.5th percentiles). Survey weights were used for the estimation of the HOPIT model. The ethnic groups in are defined as follows: "White" includes English, Welsh, Scottish, Northern Irish, British, Gypsy or Irish Traveler, and Any other White background. "Mixed or Multiple Ethnic Groups" consist of White and Black Caribbean, White and Asian, White and Black African, or Any other Mixed/Multiple ethnic background. "Asian" comprises Indian, Pakistani, Bangladeshi, Chinese, or Any other Asian ethnicity. "Black" includes African, Caribbean, or Any other Black/African/Caribbean background. "Other Ethnic Groups" include Arab or Any other ethnic group.).  ^1^Included the following explanatory variables: ethnic group, main effects of health conditions, interactions between sex and age groups and wave fixed effects. All coefficients available in the supplementary material. | | | | |

| **Table A4**. Differences in thresholds between each ethnic group and the White ethnic group baseline (Omission of area deprivation and health care utilisation variables in the threshold equations^1^) | | | | |
| --- | --- | --- | --- | --- |
| Dimension | Threshold | | | |
|  | 1\|2 | 2\|3 | 3\|4 | 4\|5 |
| Asian | | | | |
| Mobility | 0.007 (0.005) | 0.024* (0.006) | 0.033* (0.009) | 0.116* (0.019) |
| Self-Care | -0.069* (0.006) | -0.063* (0.008) | -0.17* (0.011) | -0.133* (0.017) |
| Usual Activities | 0.109* (0.004) | 0.113* (0.006) | 0.069* (0.008) | 0.094* (0.014) |
| Pain/Discomfort | -0.043* (0.003) | -0.03* (0.005) | -0.102* (0.007) | -0.063* (0.013) |
| Anxiety/Depression | 0.159* (0.003) | 0.19* (0.005) | -0.027* (0.008) | -0.079* (0.012) |
| Black | | | | |
| Mobility | -0.005 (0.007) | -0.032* (0.009) | -0.039* (0.012) | -0.053* (0.024) |
| Self-Care | -0.041* (0.009) | -0.069* (0.012) | -0.128* (0.017) | -0.105* (0.025) |
| Usual Activities | 0.147* (0.007) | 0.09* (0.009) | 0.04* (0.012) | 0.073* (0.022) |
| Pain/Discomfort | -0.041* (0.005) | -0.094* (0.007) | -0.169* (0.01) | -0.063* (0.02) |
| Anxiety/Depression | 0.279* (0.006) | 0.211* (0.008) | 0.022 (0.012) | -0.007 (0.019) |
| Mixed or multiple ethnic background | | | | |
| Mobility | -0.027* (0.011) | -0.026 (0.015) | -0.027 (0.021) | 0.135* (0.047) |
| Self-Care | -0.072* (0.014) | -0.08* (0.018) | -0.061* (0.027) | -0.056 (0.04) |
| Usual Activities | -0.021* (0.01) | 0.01 (0.015) | 0.005 (0.02) | 0.021 (0.032) |
| Pain/Discomfort | -0.064* (0.009) | -0.052* (0.012) | -0.078* (0.017) | -0.081* (0.032) |
| Anxiety/Depression | -0.054* (0.009) | -0.026* (0.011) | -0.069* (0.018) | -0.088* (0.026) |
| Other ethnic background | | | | |
| Mobility | -0.078* (0.007) | -0.086* (0.008) | -0.139* (0.011) | -0.13* (0.021) |
| Self-Care | -0.173* (0.008) | -0.16* (0.01) | -0.32* (0.013) | -0.303* (0.02) |
| Usual Activities | 0.094* (0.007) | 0.06* (0.009) | -0.041* (0.012) | -0.047* (0.017) |
| Pain/Discomfort | -0.042* (0.006) | -0.107* (0.007) | -0.261* (0.01) | -0.213* (0.016) |
| Anxiety/Depression | 0.099* (0.006) | 0.083* (0.008) | -0.218* (0.011) | -0.253* (0.015) |
|  |  |  |  |  |
| *Note.* Bootstrap standard deviations are shown in parentheses. *Indicates statistical significance at the 95% confidence level (all results were significant using both parametric bootstrap standard deviations and the 2.5th and 97.5th percentiles). Survey weights were used for the estimation of the HOPIT model. The ethnic groups in are defined as follows: "White" includes English, Welsh, Scottish, Northern Irish, British, Gypsy or Irish Traveler, and Any other White background. "Mixed or Multiple Ethnic Groups" consist of White and Black Caribbean, White and Asian, White and Black African, or Any other Mixed/Multiple ethnic background. "Asian" comprises Indian, Pakistani, Bangladeshi, Chinese, or Any other Asian ethnicity. "Black" includes African, Caribbean, or Any other Black/African/Caribbean background. "Other Ethnic Groups" include Arab or Any other ethnic group.  ^1^Included the following explanatory variables: ethnic group, main effects of health conditions, interactions between sex and age groups, wave fixed effects and two-way and three-way interactions for long-term conditions that were prevalent in at least 1% of the data. All coefficients available in the supplementary material. | | | | |

| **Table A4**. Differences in thresholds between each ethnic group and the White ethnic group baseline (age-bands and sex variables in latent equation ^1^) | | | | |
| --- | --- | --- | --- | --- |
| Dimension | Threshold | | | |
|  | 1\|2 | 2\|3 | 3\|4 | 4\|5 |
| Asian | | | | |
| Mobility | 0.104* (0.005) | 0.093* (0.007) | 0.059* (0.01) | -0.014 (0.019) |
| Self-Care | 0.031* (0.008) | 0.004 (0.01) | -0.125* (0.013) | -0.155* (0.02) |
| Usual Activities | 0.202* (0.005) | 0.181* (0.007) | 0.115* (0.01) | 0.062* (0.016) |
| Pain/Discomfort | 0.044* (0.004) | 0.043* (0.005) | -0.068* (0.008) | -0.093* (0.016) |
| Anxiety/Depression | 0.213* (0.004) | 0.243* (0.006) | 0.038* (0.01) | -0.019 (0.016) |
| Black | | | | |
| Mobility | 0.08* (0.01) | 0.014 (0.013) | -0.083* (0.019) | -0.245* (0.031) |
| Self-Care | 0.023 (0.015) | -0.041* (0.019) | -0.147* (0.025) | -0.105* (0.039) |
| Usual Activities | 0.221* (0.01) | 0.138* (0.014) | 0.018 (0.02) | 0.028 (0.031) |
| Pain/Discomfort | 0.025* (0.007) | -0.06* (0.01) | -0.173* (0.016) | -0.202* (0.029) |
| Anxiety/Depression | 0.328* (0.008) | 0.268* (0.012) | 0.088* (0.019) | -0.018 (0.031) |
| Mixed or multiple ethnic background | | | | |
| Mobility | 0.045* (0.012) | 0.02 (0.016) | -0.065* (0.024) | -0.048 (0.051) |
| Self-Care | 0.008 (0.017) | -0.028 (0.021) | -0.084* (0.032) | -0.105* (0.046) |
| Usual Activities | 0.034* (0.011) | 0.036* (0.015) | -0.017 (0.023) | -0.014 (0.035) |
| Pain/Discomfort | -0.006 (0.01) | -0.041* (0.012) | -0.117* (0.02) | -0.24* (0.034) |
| Anxiety/Depression | -0.019* (0.009) | 0 (0.011) | -0.05* (0.019) | -0.085* (0.029) |
| Other ethnic background | | | | |
| Mobility | 0.035* (0.009) | -0.008 (0.011) | -0.082* (0.015) | -0.267* (0.024) |
| Self-Care | -0.046* (0.012) | -0.068* (0.015) | -0.247* (0.018) | -0.275* (0.026) |
| Usual Activities | 0.185* (0.009) | 0.124* (0.011) | 0.000 (0.015) | -0.012 (0.022) |
| Pain/Discomfort | 0.036* (0.007) | -0.046* (0.009) | -0.226* (0.012) | -0.214* (0.022) |
| Anxiety/Depression | 0.147* (0.007) | 0.124* (0.009) | -0.149* (0.013) | -0.202* (0.022) |
|  |  |  |  |  |
| *Note.* Bootstrap standard deviations are shown in parentheses. *Indicates statistical significance at the 95% confidence level (all results were significant using both parametric bootstrap standard deviations and the 2.5th and 97.5th percentiles). Survey weights were used for the estimation of the HOPIT model. The ethnic groups in are defined as follows: "White" includes English, Welsh, Scottish, Northern Irish, British, Gypsy or Irish Traveler, and Any other White background. "Mixed or Multiple Ethnic Groups" consist of White and Black Caribbean, White and Asian, White and Black African, or Any other Mixed/Multiple ethnic background. "Asian" comprises Indian, Pakistani, Bangladeshi, Chinese, or Any other Asian ethnicity. "Black" includes African, Caribbean, or Any other Black/African/Caribbean background. "Other Ethnic Groups" include Arab or Any other ethnic group.  ^1^Included the following explanatory variables: ethnic group, main effects of health conditions, interactions between sex and age groups, wave fixed effects and two-way and three-way interactions for long-term conditions that were prevalent in at least 1% of the data. All coefficients available in the supplementary material. | | | | |

| **Table A4**. Differences in thresholds between each ethnic group and the White ethnic group baseline (area deprivation in latent equation ^1^) | | | | |
| --- | --- | --- | --- | --- |
| Dimension | Threshold | | | |
|  | 1\|2 | 2\|3 | 3\|4 | 4\|5 |
| Asian | | | | |
| Mobility | 0.098* (0.005) | 0.118* (0.006) | 0.108* (0.008) | 0.109* (0.016) |
| Self-Care | 0.038* (0.006) | 0.032* (0.007) | -0.093* (0.01) | -0.106* (0.015) |
| Usual Activities | 0.196* (0.004) | 0.199* (0.006) | 0.142* (0.008) | 0.13* (0.013) |
| Pain/Discomfort | 0.029* (0.004) | 0.067* (0.005) | -0.008 (0.008) | 0.002 (0.012) |
| Anxiety/Depression | 0.22* (0.004) | 0.259* (0.005) | 0.048* (0.008) | -0.014 (0.012) |
| Black | | | | |
| Mobility | 0.125* (0.007) | 0.091* (0.009) | 0.068* (0.012) | 0.003 (0.022) |
| Self-Care | 0.107* (0.01) | 0.065* (0.012) | -0.011 (0.016) | -0.021 (0.023) |
| Usual Activities | 0.264* (0.007) | 0.2* (0.009) | 0.136* (0.012) | 0.141* (0.019) |
| Pain/Discomfort | 0.061* (0.005) | 0.016* (0.007) | -0.072* (0.011) | 0.006 (0.019) |
| Anxiety/Depression | 0.363* (0.006) | 0.295* (0.008) | 0.102* (0.013) | 0.061* (0.02) |
| Mixed or multiple ethnic background | | | | |
| Mobility | 0.025* (0.011) | 0.027 (0.015) | 0.019 (0.021) | 0.137* (0.04) |
| Self-Care | -0.011 (0.015) | -0.022 (0.018) | -0.012 (0.027) | -0.01 (0.038) |
| Usual Activities | 0.025* (0.011) | 0.056* (0.014) | 0.046* (0.019) | 0.055. (0.029) |
| Pain/Discomfort | -0.026* (0.009) | -0.009 (0.013) | -0.039* (0.018) | -0.05 (0.03) |
| Anxiety/Depression | -0.022* (0.009) | 0.007 (0.011) | -0.033 (0.018) | -0.055* (0.025) |
| Other ethnic background | | | | |
| Mobility | 0.027* (0.007) | 0.017 (0.009) | -0.05* (0.012) | -0.085* (0.019) |
| Self-Care | -0.05* (0.009) | -0.048* (0.011) | -0.215* (0.013) | -0.23* (0.018) |
| Usual Activities | 0.191* (0.007) | 0.154* (0.009) | 0.041* (0.011) | 0.015 (0.016) |
| Pain/Discomfort | 0.039* (0.006) | -0.01 (0.007) | -0.176* (0.01) | -0.148* (0.016) |
| Anxiety/Depression | 0.168* (0.006) | 0.157* (0.008) | -0.145* (0.011) | -0.193* (0.015) |
|  |  |  |  |  |
| *Note.* Bootstrap standard deviations are shown in parentheses. *Indicates statistical significance at the 95% confidence level (all results were significant using both parametric bootstrap standard deviations and the 2.5th and 97.5th percentiles). Survey weights were used for the estimation of the HOPIT model. The ethnic groups in are defined as follows: "White" includes English, Welsh, Scottish, Northern Irish, British, Gypsy or Irish Traveler, and Any other White background. "Mixed or Multiple Ethnic Groups" consist of White and Black Caribbean, White and Asian, White and Black African, or Any other Mixed/Multiple ethnic background. "Asian" comprises Indian, Pakistani, Bangladeshi, Chinese, or Any other Asian ethnicity. "Black" includes African, Caribbean, or Any other Black/African/Caribbean background. "Other Ethnic Groups" include Arab or Any other ethnic group.  ^1^Included the following explanatory variables: ethnic group, main effects of health conditions, interactions between sex and age groups, wave fixed effects and two-way and three-way interactions for long-term conditions that were prevalent in at least 1% of the data. All coefficients available in the supplementary material. | | | | |

| **Table A4**. Differences in thresholds between each ethnic group and the White ethnic group baseline (Health care use variables in latent equation ^1^) | | | | |
| --- | --- | --- | --- | --- |
| Dimension | Threshold | | | |
|  | 1\|2 | 2\|3 | 3\|4 | 4\|5 |
| Asian | | | | |
| Mobility | 0.189* (0.005) | 0.17* (0.007) | 0.131* (0.01) | 0.045* (0.022) |
| Self-Care | 0.056* (0.007) | 0.029* (0.009) | -0.108* (0.014) | -0.136* (0.022) |
| Usual Activities | 0.24* (0.005) | 0.215* (0.007) | 0.146* (0.01) | 0.091* (0.017) |
| Pain/Discomfort | 0.109* (0.004) | 0.093* (0.005) | -0.027* (0.008) | -0.06* (0.017) |
| Anxiety/Depression | 0.181* (0.004) | 0.209* (0.006) | 0.006 (0.009) | -0.049* (0.016) |
| Black | | | | |
| Mobility | 0.156* (0.01) | 0.095* (0.013) | 0.002 (0.019) | -0.18* (0.035) |
| Self-Care | 0.049* (0.015) | -0.015 (0.018) | -0.126* (0.027) | -0.064 (0.043) |
| Usual Activities | 0.254* (0.01) | 0.171* (0.014) | 0.05* (0.02) | 0.07* (0.033) |
| Pain/Discomfort | 0.064* (0.007) | -0.029* (0.01) | -0.142* (0.015) | -0.168* (0.03) |
| Anxiety/Depression | 0.291* (0.008) | 0.231* (0.012) | 0.054* (0.019) | -0.053 (0.032) |
| Mixed or multiple ethnic background | | | | |
| Mobility | 0.154* (0.012) | 0.129* (0.017) | 0.04 (0.024) | 0.054 (0.053) |
| Self-Care | 0.051* (0.017) | 0.014 (0.022) | -0.053 (0.033) | -0.101* (0.051) |
| Usual Activities | 0.087* (0.011) | 0.088* (0.016) | 0.029 (0.023) | 0.016 (0.037) |
| Pain/Discomfort | 0.071* (0.009) | 0.029* (0.012) | -0.053* (0.019) | -0.187* (0.035) |
| Anxiety/Depression | -0.056* (0.009) | -0.037* (0.011) | -0.085* (0.019) | -0.117* (0.031) |
| Other ethnic background | | | | |
| Mobility | 0.105* (0.008) | 0.059* (0.011) | -0.022 (0.014) | -0.234* (0.028) |
| Self-Care | -0.027* (0.011) | -0.05* (0.014) | -0.247* (0.019) | -0.288* (0.029) |
| Usual Activities | 0.216* (0.009) | 0.15* (0.011) | 0.021 (0.015) | -0.001 (0.024) |
| Pain/Discomfort | 0.09* (0.007) | -0.005 (0.009) | -0.189* (0.011) | -0.188* (0.022) |
| Anxiety/Depression | 0.12* (0.007) | 0.094* (0.009) | -0.177* (0.014) | -0.232* (0.022) |
|  |  |  |  |  |
| *Note.* Bootstrap standard deviations are shown in parentheses. *Indicates statistical significance at the 95% confidence level (all results were significant using both parametric bootstrap standard deviations and the 2.5th and 97.5th percentiles). Survey weights were used for the estimation of the HOPIT model. The ethnic groups in are defined as follows: "White" includes English, Welsh, Scottish, Northern Irish, British, Gypsy or Irish Traveler, and Any other White background. "Mixed or Multiple Ethnic Groups" consist of White and Black Caribbean, White and Asian, White and Black African, or Any other Mixed/Multiple ethnic background. "Asian" comprises Indian, Pakistani, Bangladeshi, Chinese, or Any other Asian ethnicity. "Black" includes African, Caribbean, or Any other Black/African/Caribbean background. "Other Ethnic Groups" include Arab or Any other ethnic group.  ^1^Included the following explanatory variables: ethnic group, main effects of health conditions, interactions between sex and age groups, wave fixed effects and two-way and three-way interactions for long-term conditions that were prevalent in at least 1% of the data. All coefficients available in the supplementary material. | | | | |

| **Table A5.** Number and percentage of missing values by variable | | |
| --- | --- | --- |
| **Variable** | **Count** | **Percentage** |
| Last GP visit | 19,160 | 0.547 |
| Last nurse visit | 33,360 | 0.952 |
| Get appointment | 124,338 | 3.551 |
| Alzheimer’s disease or dementia | 333,716 | 9.53 |
| Angina or long-term heart problem | 333,714 | 9.53 |
| Arthritis or long-term joint problem | 333,714 | 9.53 |
| Asthma or long-term chest problem | 333,714 | 9.53 |
| Blindness or severe visual impairment | 333,714 | 9.53 |
| Cancer in the last 5 years | 333,714 | 9.53 |
| Deafness or severe hearing impairment | 333,714 | 9.53 |
| Diabetes | 333,714 | 9.53 |
| Epilepsy | 333,714 | 9.53 |
| High blood pressure | 333,714 | 9.53 |
| Kidney or liver disease | 333,714 | 9.53 |
| Long-term back problem | 333,714 | 9.53 |
| Long-term mental health problem | 333,714 | 9.53 |
| Long-term neurological problem | 333,714 | 9.53 |
| Another long-term condition | 333,714 | 9.53 |
| Mobility | 109,696 | 3.132 |
| Self-Care | 120,897 | 3.452 |
| Usual Activities | 114,278 | 3.263 |
| Pain/Discomfort | 113,716 | 3.247 |
| Anxiety/Depression | 149,936 | 4.282 |
| Activities limited today | 111,785 | 3.192 |
| Practice total size | 0 | 0 |
| Age group | 68,856 | 1.966 |
| Sex | 63,238 | 1.806 |
| Deprivation tercile | 2,310 | 0.065 |
| Ethnic group | 76,393 | 2.181 |
| *Note.* Counts and percentages are calculated based on a final sample of 3,501,404 observations. This sample was obtained after deleting observations for individuals under 25 years of age and those above 74 years of age | | |

| **Table A6:** Linear regression of missing values on demographic and health care use variables | | | | | | | | | | | | | | | |
| --- | --- | --- | --- | --- | --- | --- | --- | --- | --- | --- | --- | --- | --- | --- | --- |
| Dependent Variable:  Missing values (1 = Yes, 0 = No)^1^ | | | Model 1 | | | | Model 2 | | | | Model 3 | | | | |
| Age Group (base: 25 to 34) | | |  | | | |  | | | |  |  |  |  |  |
| 35 to 44 | | | -0.004*** (0.001) | | | | -0.001 (0.001) | | | | 0.004*** (0.001) | | | | |
| 45 to 54 | | | -0.018*** (0.001) | | | | -0.008*** (0.001) | | | | -0.001 (0.001) | | | | |
| 55 to 64 | | | -0.035*** (0.001) | | | | -0.020*** (0.001) | | | | -0.010*** (0.001) | | | | |
| 65 to 74 | | | -0.025*** (0.001) | | | | -0.006*** (0.001) | | | | 0.008*** (0.001) | | | | |
| Sex (base: female) | | |  | | | |  | | | |  | | | | |
| Male | | | -0.000 (0.001) | | | | -0.004*** (0.000) | | | | -0.018*** (0.001) | | | | |
| Deprivation Tercile (base: Least deprived) | | | |  | | | | | | | |  |  |  |  |
| Moderately Deprived | | |  | | | | 0.016*** (0.001) | | | | 0.016*** (0.001) | | | | |
| Most Deprived | | |  | | | | 0.049*** (0.001) | | | | 0.048*** (0.001) | | | | |
| Ethnic Group (base: White) | | |  | | | |  | | | |  |  |  |  |  |
| Mixed/Multiple Ethnicity | | |  | | | | 0.031*** (0.002) | | | | 0.029*** (0.002) | | | | |
| Asian | | |  | | | | 0.062*** (0.001) | | | | 0.062*** (0.001) | | | | |
| Black | | |  | | | | 0.063*** (0.001) | | | | 0.062*** (0.001) | | | | |
| Other Ethnic Group | | |  | | | | 0.160*** (0.001) | | | | 0.156*** (0.001) | | | | |
| GP Visit (base: In the past 3 months) | |  | | | |  | | | |  |  |  |  |  |  |
| Between 3 and 6 Months Ago | | |  | | | |  | | | | 0.002*** (0.001) | | | | |
| Between 6 and 12 Months Ago | | |  | | | |  | | | | 0.008*** (0.001) | | | | |
| More Than 12 Months Ago | | |  | | | |  | | | | 0.051*** (0.001) | | | | |
| Never Seen a GP | | |  | | | |  | | | | 0.302*** (0.002) | | | | |
| Nurse Visit (base: In the past 3 months) | | | |  | | | |  | | | | |  |  |  |
| Between 3 and 6 Months Ago | | |  | | | |  | | | | 0.003*** (0.001) | | | | |
| Between 6 and 12 Months Ago | | |  | | | |  | | | | 0.006*** (0.001) | | | | |
| More Than 12 Months Ago | | |  | | | |  | | | | 0.022*** (0.001) | | | | |
| Never Seen a Nurse | | |  | | | |  | | | | 0.066*** (0.001) | | | | |
| Constant | | | 0.241*** (0.001) | | | | 0.194*** (0.001) | | | | 0.162*** (0.001) | | | | |
|  |  | | | |  | | | |  | | | | |  |  |
| Observations | | | 3,416,044 | | | | 3,383,561 | | | | 3,343,609 | | | | |
| R-squared | | | 0.001 | | | | 0.011 | | | | 0.023 | | | | |
| Adjusted R-squared | | | 0.001 | | | | 0.011 | | | | 0.022 | | | | |
| Residual Std. Error | | | 0.420 (df=3,416,038) | | | | 0.413 (df=3,383,549) | | | | 0.405 (df=3,343,589) | | | | |
| F Statistic | | | 668.1***  (df=5; 3,416,038) | | | | 3,273.8***  (df=11;3,383,549) | | | | 4,051.3***  (df=19; 3,343,589) | | | | |
| Note: *p < 0.1; **p < 0.05; ***p < 0.01. Standard error in parentheses. Model fitted using weighted ordinary least squares with survey weights applied. Original dataset contained 4,377,614 observations, which were reduced to 3,501,404 after excluding individuals under 25 and above 74. Abbreviations: GP = General Practitioner.  ^1^An observation was considered to have missing values if it had at least one missing entry in the variables used in the model, excluding those used in the missing analysis model. | | | | | | | | | | | | | | |  |

| **Figure A1.** Differences in thresholds between each ethnic group and the White ethnic group for each severity level and EQ-5D domain (Omission of interactions of long-term conditions in latent equation, and area deprivation and health care use variables in the threshold equations^1^) |
| --- |
| 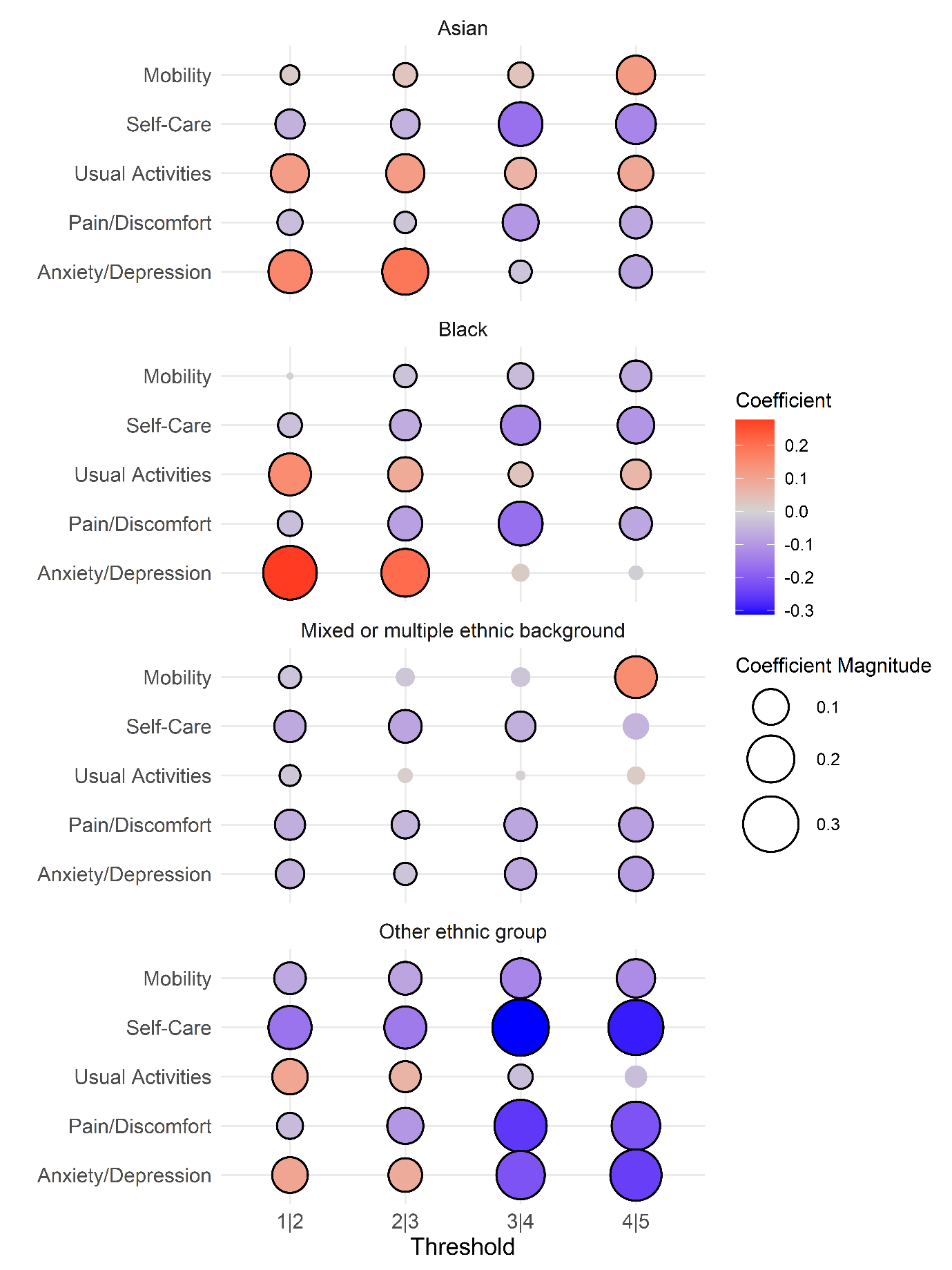 |
| *Note.* A positive value indicates greater likelihood of reporting the less severe category for the same value of the latent health index. Solid black border indicates statistical significance using percentile bootstrapping (95% CI). Survey weights were used for the estimation of the HOPIT model.  ^1^Included the following explanatory variables: ethnic group, main effects of health conditions, interactions between sex and age groups and wave fixed effects. All coefficients available in the supplementary material. |
| **Figure A2.** Differences in thresholds between each ethnic group and the White ethnic group for each severity level and EQ-5D domain (Omission of area deprivation and health care utilisation variables in the threshold equations^1^) |
| 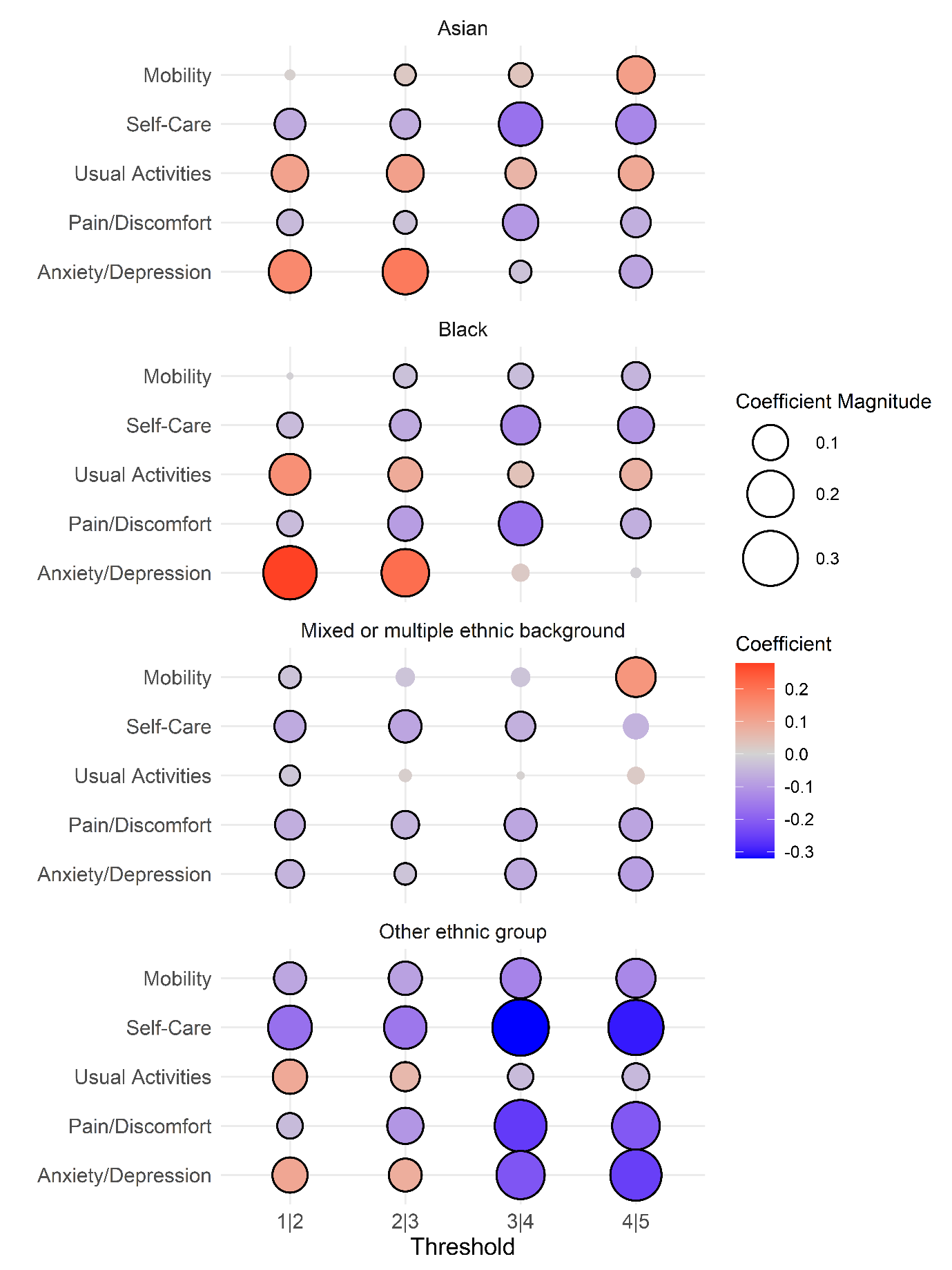 |
| *Note.* A positive value indicates greater likelihood of reporting the less severe category for the same value of the latent health index. Solid black border indicates statistical significance using percentile bootstrapping (95% CI). Survey weights were used for the estimation of the HOPIT model.  ^1^Included the following explanatory variables: ethnic group, main effects of health conditions, interactions between sex and age groups, wave fixed effects and two-way and three-way interactions for long-term conditions that were prevalent in at least 1% of the data. All coefficients available in the supplementary material. |
| **Figure A3.** Differences in thresholds between each ethnic group and the White ethnic group for each severity level and EQ-5D domain (age-bands and sex variables in latent equation^1^) |
| 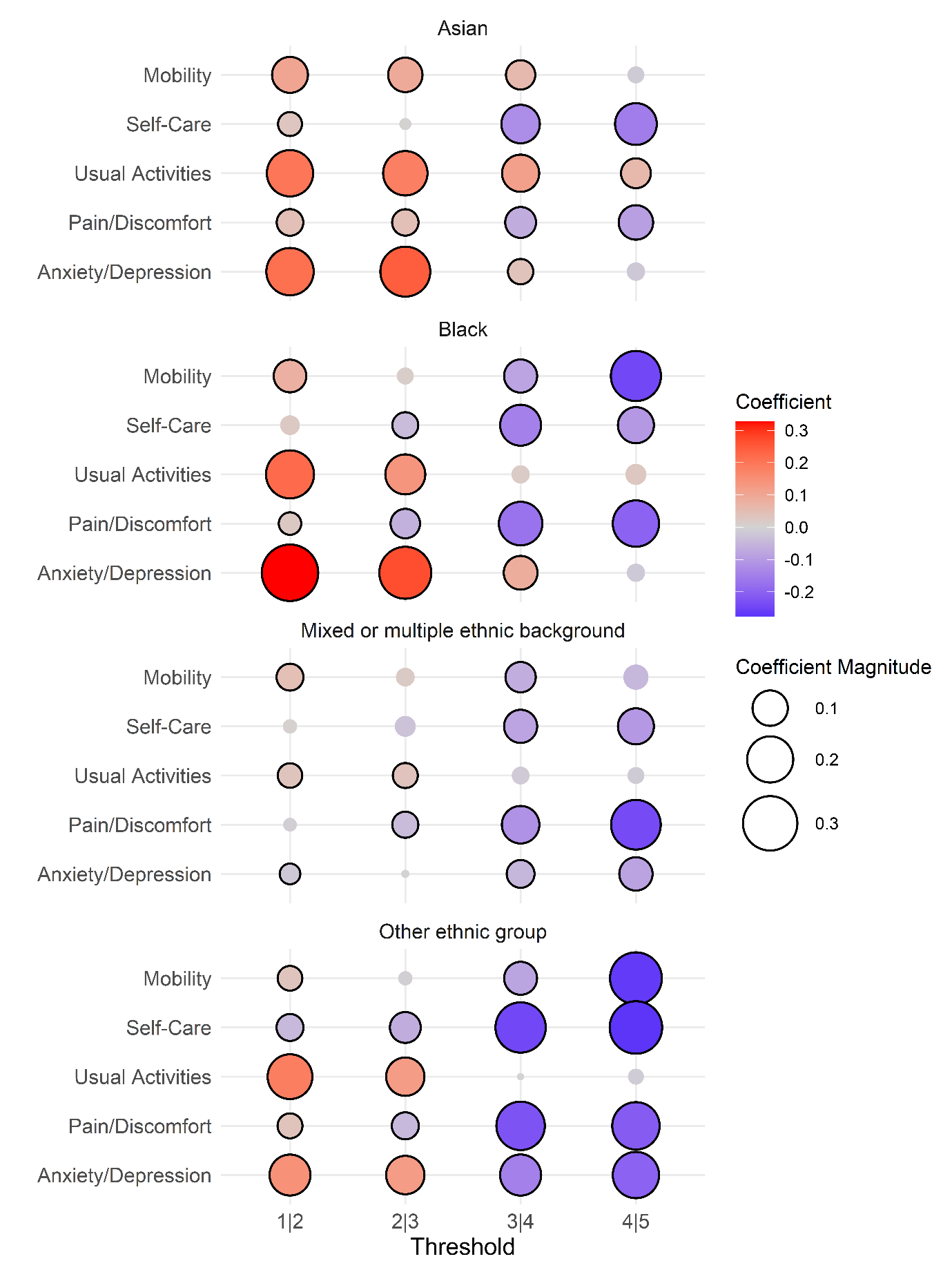 |
| *Note.* A positive value indicates greater likelihood of reporting the less severe category for the same value of the latent health index. Solid black border indicates statistical significance using percentile bootstrapping (95% CI). Survey weights were used for the estimation of the HOPIT model.  ^1^Included the following explanatory variables: ethnic group, main effects of health conditions, interactions between sex and age groups, wave fixed effects and two-way and three-way interactions for long-term conditions that were prevalent in at least 1% of the data. All coefficients available in the supplementary material. |

| **Figure A4.** Differences in thresholds between each ethnic group and the White ethnic group for each severity level and EQ-5D domain (area deprivation in latent equation^1^) |
| --- |
| 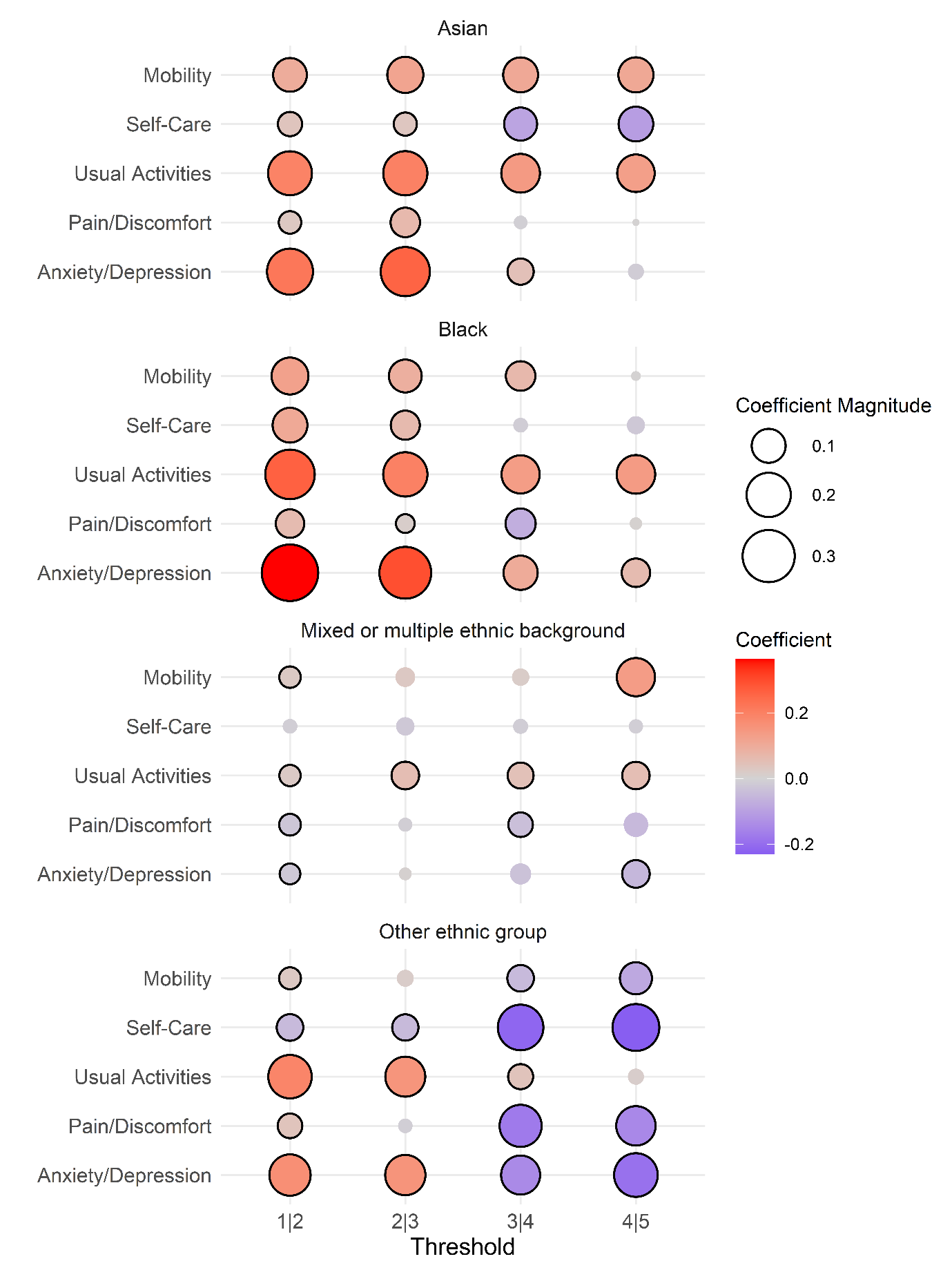 |
| *Note.* A positive value indicates greater likelihood of reporting the less severe category for the same value of the latent health index. Solid black border indicates statistical significance using percentile bootstrapping (95% CI). Survey weights were used for the estimation of the HOPIT model.  ^1^Included the following explanatory variables: ethnic group, main effects of health conditions, interactions between sex and age groups, wave fixed effects and two-way and three-way interactions for long-term conditions that were prevalent in at least 1% of the data. All coefficients available in the supplementary material. |
| **Figure A5.** Differences in thresholds between each ethnic group and the White ethnic group for each severity level and EQ-5D domain (Health care use variables in latent equation^1^) |
| 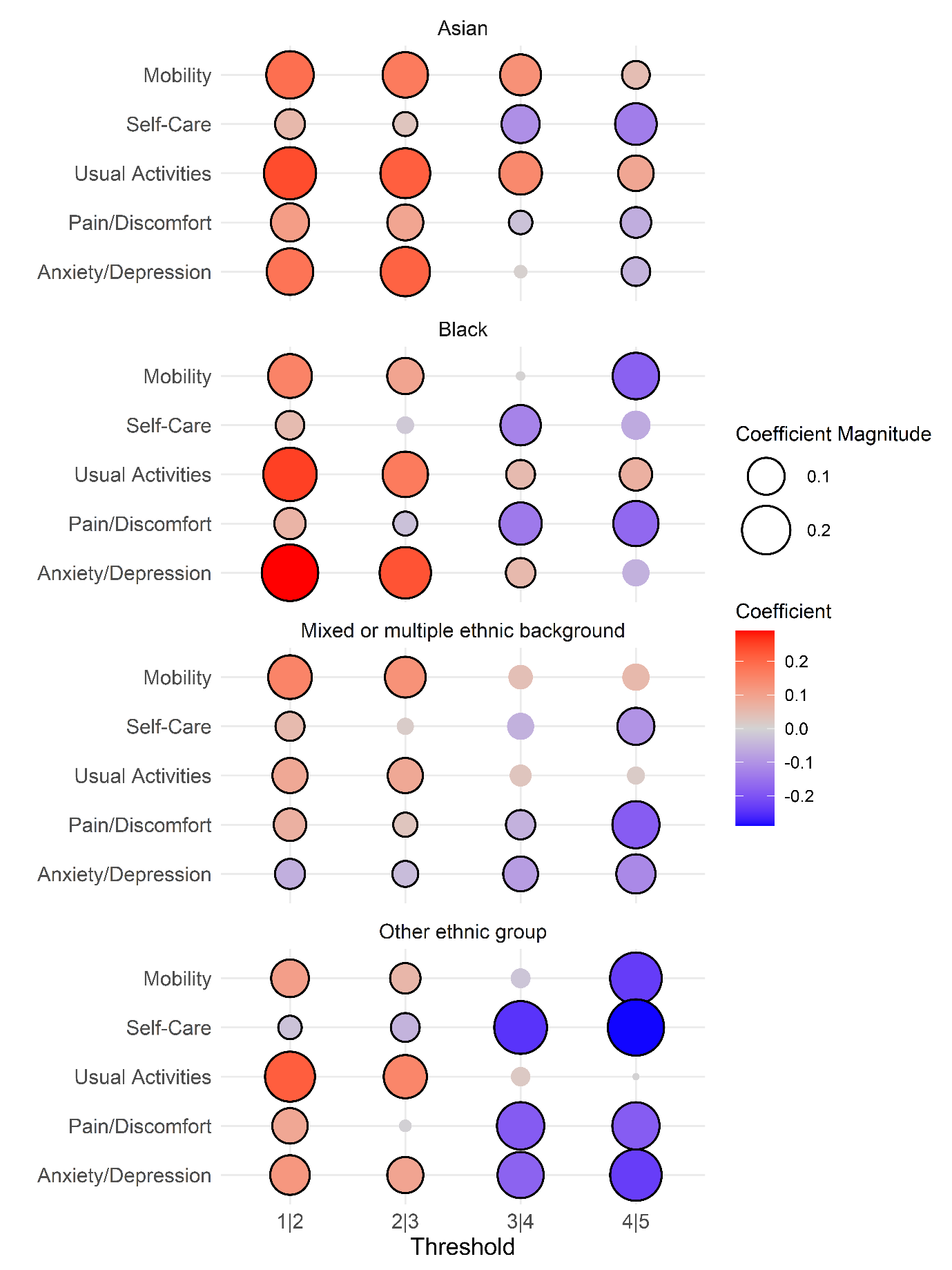 |
| *Note.* A positive value indicates greater likelihood of reporting the less severe category for the same value of the latent health index. Solid black border indicates statistical significance using percentile bootstrapping (95% CI). Survey weights were used for the estimation of the HOPIT model. |

| **Table A7.** Differences in thresholds between each ethnic group within the White ethnic group and English/Welsh/Scottish/Nothern/Irish/British group baseline | | | | |
| --- | --- | --- | --- | --- |
| Dimension | Thresholds between each of the levels | | | |
|  | 1\|2 | 2\|3 | 3\|4 | 4\|5 |
| Irish | | | | |
| Mobility | 0.091* (0.012) | 0.024 (0.014) | -0.004 (0.018) | 0.008 (0.032) |
| Self-Care | -0.026 (0.014) | -0.047* (0.017) | -0.058* (0.024) | 0.011 (0.037) |
| Usual Activities | 0.099* (0.011) | 0.002 (0.014) | -0.008 (0.018) | -0.025 (0.024) |
| Pain/Discomfort | 0.109* (0.01) | -0.001 (0.011) | -0.019 (0.017) | 0.017 (0.03) |
| Anxiety/Depression | 0.042* (0.01) | 0.017 (0.013) | 0.021 (0.021) | 0.039 (0.03) |
| Gypsy or Irish Traveler | | | | |
| Mobility | -0.274* (0.054) | -0.432* (0.06) | -0.342* (0.072) | -0.055 (0.115) |
| Self-Care | -0.508* (0.058) | -0.506* (0.063) | -0.383* (0.081) | -0.241~ (0.113) |
| Usual Activities | -0.36* (0.053) | -0.408* (0.06) | -0.326* (0.068) | -0.161 (0.094) |
| Pain/Discomfort | -0.162* (0.052) | -0.465* (0.054) | -0.592* (0.065) | -0.464* (0.092) |
| Anxiety/Depression | -0.124* (0.049) | -0.388* (0.055) | -0.623* (0.067) | -0.557* (0.083) |
| Any other White background | | | | |
| Mobility | 0.174* (0.005) | 0.164* (0.007) | 0.109* (0.01) | -0.041* (0.016) |
| Self-Care | 0.171* (0.007) | 0.151* (0.009) | -0.033* (0.012) | -0.118* (0.016) |
| Usual Activities | 0.172* (0.005) | 0.198* (0.007) | 0.144* (0.009) | 0.059* (0.013) |
| Pain/Discomfort | -0.025* (0.004) | 0.046* (0.005) | 0.018* (0.008) | 0.048* (0.014) |
| Anxiety/Depression | 0.13* (0.004) | 0.17* (0.005) | 0.05* (0.008) | 0.058* (0.013) |
| Note. Bootstrap standard deviations are shown in parentheses. *Indicates statistical significance at the 95% confidence level using both parametric bootstrap standard deviations and the 2.5th and 97.5th percentiles). ~Indicates statistical significance at the 95% confidence level using standard deviations). | | | | |

| **Table A8.** Differences in thresholds between each ethnic group within the Mixed or multiple ethnic background group and English/Welsh/Scottish/Nothern/Irish/British group baseline | | | | |
| --- | --- | --- | --- | --- |
| Dimension | Thresholds between each of the levels | | | |
|  | 1\|2 | 2\|3 | 3\|4 | 4\|5 |
| White and Black Caribbean | | | | |
| Mobility | -0.067* (0.02) | -0.072* (0.027) | -0.036 (0.037) | -0.017 (0.066) |
| Self-Care | -0.047 (0.027) | -0.025 (0.033) | -0.046 (0.045) | -0.076 (0.063) |
| Usual Activities | -0.034 (0.019) | 0.011 (0.027) | -0.015 (0.035) | -0.058 (0.05) |
| Pain/Discomfort | -0.073* (0.017) | -0.044 (0.024) | -0.144* (0.031) | -0.112* (0.053) |
| Anxiety/Depression | -0.041* (0.016) | -0.022 (0.022) | -0.02 (0.033) | 0.032 (0.046) |
| White and Asian | | | | |
| Mobility | 0.111* (0.022) | 0.145* (0.031) | 0.036 (0.04) | 0.005 (0.069) |
| Self-Care | 0.02 (0.03) | 0.037 (0.036) | -0.031 (0.048) | -0.035 (0.076) |
| Usual Activities | 0.1* (0.02) | 0.126* (0.027) | 0.149* (0.04) | 0.153* (0.059) |
| Pain/Discomfort | 0.007 (0.017) | 0.073* (0.024) | 0.055 (0.037) | -0.016 (0.059) |
| Anxiety/Depression | 0.003 (0.016) | 0.096* (0.023) | 0.094* (0.036) | 0.052 (0.052) |
| White and Black African | | | | |
| Mobility | 0.143* (0.03) | 0.057 (0.038) | 0.086 (0.054) | 0.255* (0.11) |
| Self-Care | 0.051 (0.037) | -0.023 (0.043) | 0.026 (0.063) | -0.085 (0.082) |
| Usual Activities | 0.173* (0.027) | 0.14* (0.038) | 0.159* (0.051) | 0.18* (0.08) |
| Pain/Discomfort | 0.043. (0.022) | 0.015 (0.03) | -0.043 (0.045) | 0.214* (0.086) |
| Anxiety/Depression | 0.079* (0.022) | 0.087* (0.03) | 0.004 (0.047) | 0.018 (0.065) |
| Any other Mixed/Multiple ethnic background | | | | |
| Mobility | 0.034 (0.021) | 0.076* (0.028) | 0.051 (0.04) | 0.173* (0.073) |
| Self-Care | 0.018 (0.027) | -0.03 (0.032) | -0.028 (0.045) | -0.042 (0.06) |
| Usual Activities | -0.009 (0.019) | 0.068* (0.026) | 0.01 (0.033) | 0.001 (0.047) |
| Pain/Discomfort | -0.082* (0.016) | -0.019 (0.022) | 0.039 (0.034) | -0.094 (0.05) |
| Anxiety/Depression | -0.047* (0.016) | -0.012 (0.021) | -0.099* (0.031) | -0.187* (0.042) |
| Note. Bootstrap standard deviations are shown in parentheses. *Indicates statistical significance at the 95% confidence level using both parametric bootstrap standard deviations and the 2.5th and 97.5th percentiles). ~Indicates statistical significance at the 95% confidence level using standard deviations). | | | | |

| **Table A9.** Differences in thresholds between each ethnic group within the Asian ethnic group and English/Welsh/Scottish/Nothern/Irish/British group baseline | | | | |
| --- | --- | --- | --- | --- |
| Dimension | Thresholds between each of the levels | | | |
|  | 1\|2 | 2\|3 | 3\|4 | 4\|5 |
| Indian | | | | |
| Mobility | 0.142* (0.008) | 0.122* (0.01) | 0.101* (0.013) | 0.065* (0.024) |
| Self-Care | 0.099* (0.01) | 0.057* (0.012) | -0.062* (0.016) | -0.123* (0.022) |
| Usual Activities | 0.26* (0.007) | 0.238* (0.01) | 0.192* (0.014) | 0.138* (0.021) |
| Pain/Discomfort | 0.101* (0.006) | 0.1* (0.008) | 0.068* (0.012) | 0.063* (0.021) |
| Anxiety/Depression | 0.306* (0.006) | 0.321* (0.009) | 0.157* (0.015) | 0.095* (0.022) |
| Pakistani | | | | |
| Mobility | 0.03* (0.009) | 0.049* (0.012) | 0.058* (0.015) | 0.021 (0.026) |
| Self-Care | -0.065* (0.012) | -0.043* (0.014) | -0.182* (0.017) | -0.199* (0.023) |
| Usual Activities | 0.087* (0.008) | 0.105* (0.012) | 0.07* (0.015) | 0.055* (0.021) |
| Pain/Discomfort | -0.037* (0.007) | -0.029* (0.01) | -0.087* (0.013) | -0.044~ (0.022) |
| Anxiety/Depression | 0.197* (0.007) | 0.229* (0.01) | 0.004 (0.015) | -0.082* (0.021) |
| Bangladeshi | | | | |
| Mobility | -0.007 (0.015) | 0.136* (0.021) | 0.198* (0.027) | 0.236* (0.053) |
| Self-Care | -0.043* (0.018) | 0.031 (0.022) | -0.136* (0.027) | -0.185* (0.037) |
| Usual Activities | 0.061* (0.014) | 0.173* (0.019) | 0.136* (0.026) | 0.178* (0.038) |
| Pain/Discomfort | -0.179* (0.012) | -0.009 (0.017) | -0.036 (0.023) | -0.006 (0.036) |
| Anxiety/Depression | 0.145* (0.012) | 0.261* (0.017) | -0.003 (0.024) | -0.074* (0.034) |
| Chinese | | | | |
| Mobility | 0.384* (0.017) | 0.472* (0.027) | 0.394* (0.041) | -0.014 (0.065) |
| Self-Care | 0.441* (0.029) | 0.403* (0.039) | 0.181* (0.055) | 0.112 (0.084) |
| Usual Activities | 0.514* (0.016) | 0.582* (0.027) | 0.448* (0.043) | 0.373* (0.069) |
| Pain/Discomfort | 0.034* (0.011) | 0.315* (0.019) | 0.335* (0.034) | 0.138* (0.06) |
| Anxiety/Depression | 0.093* (0.011) | 0.321* (0.018) | 0.235* (0.034) | 0.362* (0.066) |
| Any other Asian background | | | | |
| Mobility | 0.121* (0.01) | 0.174* (0.013) | 0.111* (0.017) | 0.078* (0.03) |
| Self-Care | 0.077* (0.013) | 0.057* (0.016) | -0.082* (0.021) | -0.126* (0.027) |
| Usual Activities | 0.228* (0.009) | 0.255* (0.013) | 0.13* (0.017) | 0.096* (0.025) |
| Pain/Discomfort | -0.004 (0.008) | 0.125* (0.011) | -0.037* (0.015) | -0.051~ (0.025) |
| Anxiety/Depression | 0.233* (0.007) | 0.275* (0.011) | -0.003 (0.016) | -0.055* (0.024) |
| Note. Bootstrap standard deviations are shown in parentheses. *Indicates statistical significance at the 95% confidence level using both parametric bootstrap standard deviations and the 2.5th and 97.5th percentiles). ~Indicates statistical significance at the 95% confidence level using standard deviations). | | | | |

| **Table A10.** Differences in thresholds between each ethnic group within the Black ethnic group and English/Welsh/Scottish/Nothern/Irish/British group baseline | | | | |
| --- | --- | --- | --- | --- |
| Dimension | Thresholds between each of the levels | | | |
|  | 1\|2 | 2\|3 | 3\|4 | 4\|5 |
| African | | | | |
| Mobility | 0.244* (0.009) | 0.155* (0.014) | 0.061* (0.017) | -0.05 (0.03) |
| Self-Care | 0.22* (0.013) | 0.132* (0.017) | -0.041~ (0.02) | -0.099* (0.029) |
| Usual Activities | 0.387* (0.01) | 0.3* (0.014) | 0.164* (0.018) | 0.113* (0.028) |
| Pain/Discomfort | 0.106* (0.007) | 0.097* (0.01) | -0.057* (0.015) | 0.036 (0.027) |
| Anxiety/Depression | 0.476* (0.008) | 0.424* (0.012) | 0.186* (0.018) | 0.15* (0.028) |
| Caribbean | | | | |
| Mobility | 0.035* (0.012) | 0.084* (0.016) | 0.116* (0.021) | -0.07* (0.032) |
| Self-Care | 0.054* (0.016) | 0.051* (0.019) | 0.027 (0.026) | -0.081* (0.035) |
| Usual Activities | 0.155* (0.012) | 0.156* (0.015) | 0.138* (0.021) | 0.107* (0.032) |
| Pain/Discomfort | -0.068* (0.01) | -0.011 (0.013) | 0.024 (0.018) | 0.046 (0.031) |
| Anxiety/Depression | 0.229* (0.01) | 0.234* (0.014) | 0.148* (0.023) | 0.102* (0.032) |
| Any other Black/African/Caribbean background | | | | |
| Mobility | 0.021 (0.016) | 0.033 (0.021) | 0.012 (0.028) | -0.06 (0.044) |
| Self-Care | -0.004 (0.022) | -0.03 (0.026) | -0.093* (0.034) | -0.041 (0.049) |
| Usual Activities | 0.166* (0.017) | 0.141* (0.022) | 0.098* (0.028) | 0.131* (0.041) |
| Pain/Discomfort | -0.029* (0.014) | -0.057* (0.018) | -0.119* (0.025) | -0.104* (0.04) |
| Anxiety/Depression | 0.238* (0.015) | 0.163* (0.018) | -0.029 (0.028) | -0.088* (0.039) |
| Note. Bootstrap standard deviations are shown in parentheses. *Indicates statistical significance at the 95% confidence level using both parametric bootstrap standard deviations and the 2.5th and 97.5th percentiles). ~Indicates statistical significance at the 95% confidence level using standard deviations). | | | | |

| **Table A11.** Differences in thresholds between each ethnic group within the Other ethnic group and English/Welsh/Scottish/Nothern/Irish/British group baseline | | | | |
| --- | --- | --- | --- | --- |
| Dimension | Thresholds between each of the levels | | | |
|  | 1\|2 | 2\|3 | 3\|4 | 4\|5 |
| Arab | | | | |
| Mobility | 0 (0.023) | 0.045 (0.03) | 0.031 (0.04) | 0.057 (0.063) |
| Self-Care | -0.066* (0.028) | -0.099* (0.032) | -0.244* (0.039) | -0.175* (0.056) |
| Usual Activities | 0.1* (0.022) | 0.08* (0.028) | 0.014 (0.037) | 0.138* (0.052) |
| Pain/Discomfort | -0.032 (0.02) | -0.146* (0.024) | -0.088* (0.035) | -0.079 (0.048) |
| Anxiety/Depression | 0.049* (0.019) | 0.017 (0.024) | -0.233* (0.033) | -0.146* (0.048) |
| Any other ethnic group | | | | |
| Mobility | 0.049* (0.007) | 0.038* (0.009) | -0.047* (0.012) | -0.151* (0.018) |
| Self-Care | -0.029* (0.009) | -0.03* (0.011) | -0.218* (0.013) | -0.279* (0.017) |
| Usual Activities | 0.216* (0.007) | 0.188* (0.01) | 0.058* (0.012) | -0.014 (0.015) |
| Pain/Discomfort | 0.028* (0.006) | 0.022* (0.008) | -0.162* (0.01) | -0.152* (0.016) |
| Anxiety/Depression | 0.188* (0.006) | 0.2* (0.008) | -0.11* (0.011) | -0.181* (0.016) |
| Note. Bootstrap standard deviations are shown in parentheses. *Indicates statistical significance at the 95% confidence level using both parametric bootstrap standard deviations and the 2.5th and 97.5th percentiles). ~Indicates statistical significance at the 95% confidence level using standard deviations). | | | | |
